# Comparative Safety and Efficacy of Patient-Specific Versus Hand-Molded Implants in Cranioplasty: A Systematic Review and Meta-Analysis

**DOI:** 10.3390/jcm14248655

**Published:** 2025-12-06

**Authors:** Elias-Leon Nolden, Bruna Katherine Guimarães Carvalho, Katarina Sofia Barkovskij-Jakobsen, Alexander Schulze Wenning, Boglárka Lilla Szentes, Gergely Agócs, Zsolt Németh, Márton Kivovics, Péter Hegyi, László Köles, Mihály Vaszilkó

**Affiliations:** 1 Centre for Translational Medicine, Semmelweis University, 1085 Budapest, Hungary; 2 Department of Oral Biology, Semmelweis University, 1089 Budapest, Hungary; 3 Department of Oro-Maxillofacial Surgery and Stomatology, Semmelweis University, 1085 Budapest, Hungary; 4 Department of Biophysics and Radiation Biology, Semmelweis University, 1094 Budapest, Hungary; 5 Department of Public Dental Health, Semmelweis University, 1088 Budapest, Hungary; 6 Institute of Pancreatic Diseases, Semmelweis University, 1083 Budapest, Hungary; 7 Institute for Translational Medicine, Medical School, University of Pécs, 7624 Pécs, Hungary

**Keywords:** cranioplasty, cranial reconstruction, prostheses and implants, maxillofacial surgery

## Abstract

**Background/Objectives:** Cranioplasty (CP) is associated with high complication rates (20–50%), and the optimal choice between patient-specific implants (PSIs) and hand-molded (HM) alternatives remains debated. This systematic review and meta-analysis aims to compare surgical and postoperative outcomes between PSIs and HM implants. **Methods:** A systematic search was performed in three databases to identify studies reporting surgical site infection (SSI), implant removal, reoperation, operative time or cosmetic outcome for PSIs and/or HM implants. Two-arm studies of the same material were analyzed separately from pooled single- and two-arm studies. **Results:** 125 observational studies involving 10,034 patients were included. In two-arm comparisons, PSIs reduced implant removal for titanium (OR 0.34, *p* = 0.053) and PMMA (OR 0.56, *p* = 0.188), while SSI rates showed no meaningful difference between groups. In one-arm analyses, PSIs demonstrated lower explantation probabilities (titanium 6.1%, PMMA 7.9%) compared with HM alternatives (titanium 9.9%, PMMA 14.2%), alongside shorter operation times and fewer reoperations. Cosmetic outcomes consistently favored PSIs. **Conclusions:** PSIs demonstrate advantages in efficiency, durability, and esthetics compared with HM implants, supporting their preferential use where resources allow. HM implants remain a cost-effective option in resource-limited settings. Due to the observational nature of the included studies and differences in study populations across arms, the findings should be interpreted with caution.

## 1. Introduction

Cranioplasty (CP) is a surgical procedure performed to repair cranial defects, most commonly following decompressive craniectomy, which is used as a life-saving intervention in cases of traumatic brain injury, stroke, or intracranial hypertension [1]. Beyond restoring skull integrity and protecting the underlying brain, CP also contributes to improved cerebral hemodynamics and neurological function, as well as providing critical cosmetic reconstruction, which can greatly affect patient self-image and quality of life [2]. As a result, timely and effective CP is now regarded as a critical component in supporting neurological recovery and overall rehabilitation. The growing use of CP in both emergency and elective neurosurgical procedures has led to a rising clinical demand for better implant solutions [3].

However, CP is not without risks. Despite being a reconstructive and life-saving operation, CP carries an unusually high complication rate ranging from 20 to 50%–higher than most other neurosurgical procedures [3,4]. Complications include surgical site infection (SSI), implant extrusion, wound breakdown, and the need for revision surgeries, which often come with prolonged operation time and poor cosmetic outcome. These risks are strongly influenced by multiple factors, including the material used, the timing of surgery, and the precision of implant fit [5].

To address these challenges, the use of patient-specific implants (PSIs)–fully customized prostheses designed using 3D printing and computer-aided design and computer-aided manufacturing technology–has gained momentum. Materials commonly used in PSIs include titanium, polyetheretherketone (PEEK), and polymethylmethacrylate (PMMA), each with distinct biological, mechanical, and clinical profiles [6,7,8,9].

Despite these developments, there remains no clear consensus on the optimal implant material or the superiority of PSIs over traditional techniques [10,11]. Current literature is fragmented, often limited to small, retrospective series with heterogeneous patient populations and inconsistent outcome reporting. Prior reviews have largely grouped synthetic materials together or failed to isolate fully customized PSIs for head-to-head comparison across critical endpoints like SSI, implant failure and cosmetic success [4,12,13]. Given the increasing adoption of patient-specific technology, the rising demand for CP and the lack of high-level comparative data, an updated systematic review and meta-analysis is urgently needed.

The primary aim of this study is to systematically evaluate the current evidence regarding the use of fully customized PSIs in CP. We aim to compare postoperative outcomes, including SSI, implant failure, total reoperation rate, operation time, and cosmetic results across these materials. Through a meta-analytic approach, we seek to clarify whether the type of PSI material influences complication rates and patient outcomes and to guide future clinical decision-making and standardization in CP practices.

## 2. Materials and Methods

This systematic review and meta-analysis was conducted in accordance with the PRISMA 2020 guidelines [14] and adhered to the methodology outlined in the Cochrane Handbook for Systematic Reviews of Interventions [15] (Supplementary Material Table S1). The review protocol was registered prospectively with PROSPERO (registration number: CRD42024582985). This work was carried out as part of the Systems Education Program [16].

### 2.1. Eligibility Criteria

The research question was structured using the PICO framework, where the population (P) consisted of patients undergoing CP, the intervention (I) was the use of PSI, the comparison (C) was the use of intraoperative HM implants, and the outcomes (O) were postoperative complications (SSI, implant failure, total reoperation rate), operation time, cosmetic score and implant cost. To be eligible, studies had to involve human participants who received a CP using either a PSI or a HM implant and had to report on at least one of the predefined outcomes. No restriction was placed on the length of follow-up time to ensure comprehensive inclusion of outcome data. However, studies exclusively involving pediatric populations or mixed adult-pediatric cohorts were excluded to reduce demographic heterogeneity. Eligible studies included both comparative (two-arm) and single-arm observational cohorts. Two-arm studies were defined as those comparing PSIs and HM implants made of the same material within the same study, and these contributed to direct comparisons (odds ratios (OR)). Single-arm studies were defined as those reporting only one implant type and/or multiple materials without a direct PSI–HM comparison, and these contributed to pooled proportions and meta-regression analyses. Eligible articles needed to report raw outcome data for at least one of the prespecified endpoints. Case reports, small case series, conference abstracts, and studies lacking original data were excluded. Where multiple studies reported on the same patient cohort, only the publication with the largest sample size was included.

### 2.2. Selection Process and Search Strategy

A comprehensive literature search was carried out on 25 August 2024, using three electronic databases: MEDLINE (via PubMed), Embase, and the Cochrane Central Register of Controlled Trials (CENTRAL). The search was conducted without any limitations on publication date or study type. Although language restrictions were not applied during the initial search, studies unavailable in English, German, or Hungarian were excluded during full-text screening. The complete search strategy is outlined in Supplementary Material (Supplementary Material Text S1). Three reviewers (ELN, BKGC and KSBJ) independently screened titles and abstracts, followed by full-text assessment for final inclusion. Duplicate entries were removed both automatically and manually. Any disagreement between reviewers was resolved through consensus. Inter-rater agreement was assessed using Cohen’s kappa, yielding high concordance scores (κ = 0.96 for abstract screening and κ = 0.97 for full-text selection).

### 2.3. Data Collection Process and Extracted Variables

Three reviewers (ELN, BKGC, and KSBJ) independently extracted data from all eligible studies. Any discrepancies were resolved through discussion to reach consensus. The extracted information included: (1) study details—such as first author, publication year, design, study population (sample size, age, and sex assigned at birth), study period, country, institution, diagnosis, implant type, and follow-up duration (in months); (2) postoperative complications; (3) operative time; and (4) cosmetic outcomes. In this study, outcome definitions were standardized across all included articles. Outcome definitions varied across studies. SSI was defined as any postoperative infection involving the incision, soft tissues, or deeper cranial compartments related to the cranioplasty procedure. This included superficial wound infections, deep infections involving soft tissues or bone, and intracranial or organ-space infections when explicitly attributed to the implant or surgical site. These categories were harmonized under a unified SSI outcome to enable consistent extraction across studies. Implant failure was defined as the removal or revision of the implant due to postoperative complications. Reoperation was defined as any subsequent ambulatory or surgical intervention following the initial implantation. Implant removal and total reoperation were analyzed separately to capture the full burden of complications, as “implant removal” reflects material- or implant-specific failure, while “reoperation” includes wound revisions or soft-tissue procedures not necessarily requiring explantation. Operation time was measured from the initial skin incision to the final closure of the surgical site. Cosmetic outcomes were reported using a VAS ranging from 0 to 10, with 10 representing the best possible esthetic result and 0 the poorest.

### 2.4. Assessment of the Risk of Bias and Certainty of the Evidence

The risk of bias in the included studies was independently assessed by three reviewers (ELN, BKGC, and KSBJ) using the RoB 2 tool for randomized studies, the ROBINS-I tool and the methodological index for non-randomized studies (MINORS) [15,17]. Any discrepancies in scoring were resolved through discussion. The certainty of evidence across outcomes was rated using the GRADE framework, in line with the GRADE handbook [18], and processed using GRADEpro GDT software (version 2013) [19].

### 2.5. Synthesis Methods

This meta-analysis investigated the differences in various outcomes with PSIs and HM implants in patients with CP. Data for 6 different outcomes were available in the studies: 3 continuous and 3 dichotomous. The main focus was on the different design and material combinations, from which we could identify 10 different ones. Since not every material allows for both PSI and HM design, direct comparisons across methods were not always feasible. For designs where a direct same-material comparison between PSIs and HM implants was possible, the corresponding two-arm studies were analyzed separately using OR for dichotomous outcomes. All single-arm studies lacking direct comparators, were synthesized in pooled proportion analyses and meta-regression using a multilevel random-effects model. To calculate the proportion or the OR, sample size and number of events were extracted from the manuscript. OR were reported as the odds of the event in the PSI group against the odds of the event in the HM group. Otherwise, proportions and mean values were calculated, respectively. Proportions were logit transformed before running the meta regression using the escalc() function. Continuity correction of 0.5 for 0 or 100% proportions was applied and the corresponding sample variance was used with a diagonal variance-covariance matrix. This error structure implies the statistical independence of sampling errors, which can be justified since there are no shared controls, repeated measures, or one patient cannot get multiple different treatments. Material and design were used as a combined factor variable using every combination as a single level. Two outcomes needed data modifications. Implant prices extracted from the studies in their original currency, exchanged for USD at the exchange rate from the middle of the study period, were inflation-adjusted but were not pooled. Cosmetic scores were pooled, but because of the differing scales used, the scales were converted to a 0 to 10 scale before the analysis. Some studies reported more than one results for the same outcomes for different material/design combinations. Although the studies reported average measurement values and standard errors corresponding to distinct combinations, i.e., the correlations among the within-study error terms can be assumed to be 0; the random-effect terms within a single study are correlated when a study contributes to the pooled results with more than one measurement result. For this reason, to calculate pooled results we used multivariate meta-analysis with the rma.mv() function of the metafor R package (version 4.8.0). A two-level hierarchical structure was employed for the random effect terms. We assumed that effect sizes are nested within studies. Statistical analysis was conducted using R version 4.4.3 (R Core Team, R Foundation for Statistical Computing, Vienna, Austria) based on the recommendations from Harrer et al. [20]. Absolute between-study heterogeneity was expressed by tau, and relative between-study heterogeneity was described by Higgins and Thompson’s I squared statistics [21].

## 3. Results

### 3.1. Search and Selection

**Figure 1:**
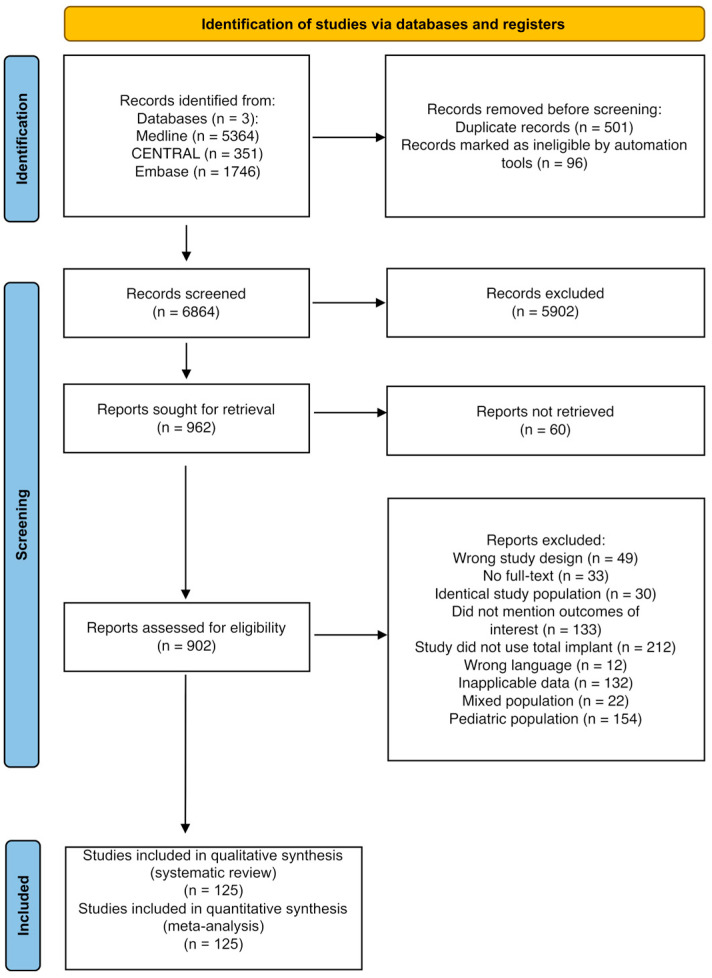
PRISMA flowchart of the study selection process.

Of the 7461 articles screened, 902 underwent full-text review, of which 125 met the inclusion criteria (Figure 1). A total of 98 studies evaluated PSI, and 69 evaluated HM implants, with several studies assessing both. Data from 10,034 patients were analyzed, with 6170 in the PSI group and 3864 in the HM implant group. Baseline characteristics are detailed in Table 1.

### 3.2. Risk of Bias Assessment

Single-arm studies were evaluated using the MINORS scale, with most demonstrating a low to moderate risk of bias, mainly due to the lack of blinded assessments and the absence of prospective sample size calculations. Several studies also reported follow-up losses exceeding 5%. Among the ten two-arm studies, the ROBINS-I tool identified a generally moderate risk of bias, with some studies showing serious risks related to confounding factors, subjective outcome measurements, and missing data. The single randomized controlled trial was assessed using the ROB2 tool and rated as having some concerns due to missing outcome data. Detailed results are provided in Supplementary Material (Supplementary Material Table S2 and Figures S1 and S2).

### 3.3. Implant Removal

**Figure 2:**
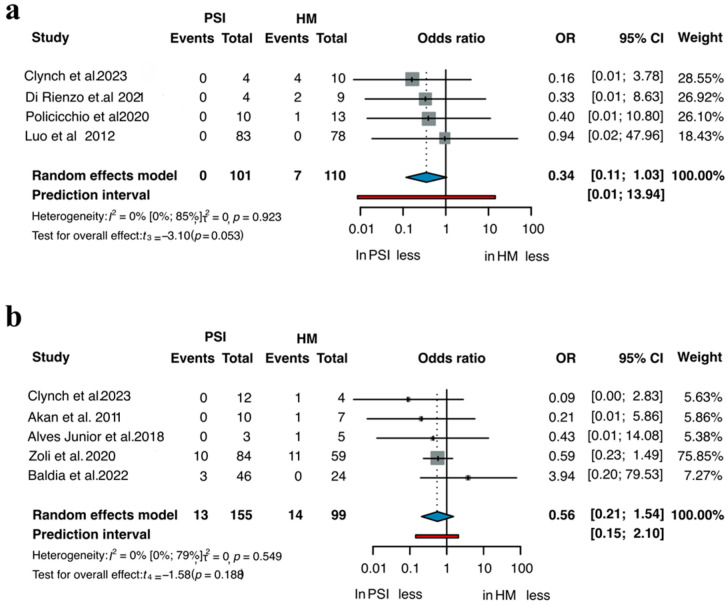
Removal of (**a**) Titanium and (**b**) PMMA implants across all two-arm studies [3,22,28,40,45,83,102,138].

**Figure 3:**
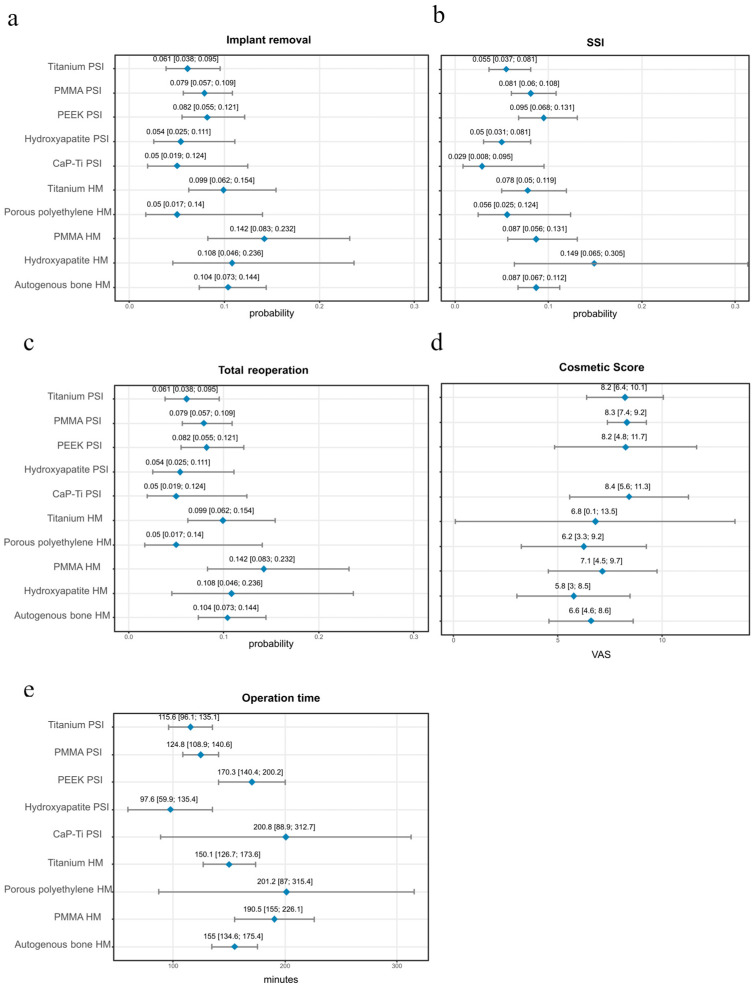
Visualized Meta-Regression estimates with 95% CI for implant materials. None of the single-arm analyses reached statistical significance. (**a**) Implant removal; (**b**) SSI; (**c**) Total reoperation; (**d**) Cosmetic Score; (**e**) Operation time. PSI: Patient-Specific Implant, HM: Hand-molded, SSI: Surgical Side Infection, CI: Confidence Interval, VAS: Visual Analogue Scale, PMMA: Polymethylmethacrylate, PEEK: Polyetheretherketone, CI: Confidence Interval.

A total of eight comparative two-arm studies allowed for the evaluation of implant removal rates between PSIs and HM implants [3,22,28,40,45,83,102,138]. Separate subgroup analyses were performed for titanium and PMMA implants, as illustrated in Figure 2. Across studies including 101 patients treated with PSIs and 110 patients with HM titanium implants, the OR for implant removal was 0.34 in favor of PSIs (95% CI 0.11–1.03; *p* = 0.053). In studies involving 155 patients in the PMMA PSI group and 99 in the HM group, the OR was 0.56 (95% CI 0.21–1.54; *p* = 0.188), again indicating a trend toward fewer removals in the PSI group, though not statistically significant. Table S3 summarizes the results of the one arm analyses for implant removal, showing that all PSI materials demonstrated lower probabilities of explantation compared to HM implants of the same material. Across all included studies, PSIs consistently reduced the risk of postoperative removal, with titanium and PMMA PSIs showing lower rates than their HM counterparts, with the strongest effect being observed for titanium (Figure 3a). In pooled analyses, PSI materials such as CaP-titanium and hydroxyapatite exhibited the lowest explantation rates (<6%), whereas HM PMMA showed the highest rate (14.2%). These findings indicate a consistent advantage of PSIs across materials, although statistical significance was not uniformly achieved in the two-arm subgroup comparisons.

### 3.4. SSI

**Figure 4:**
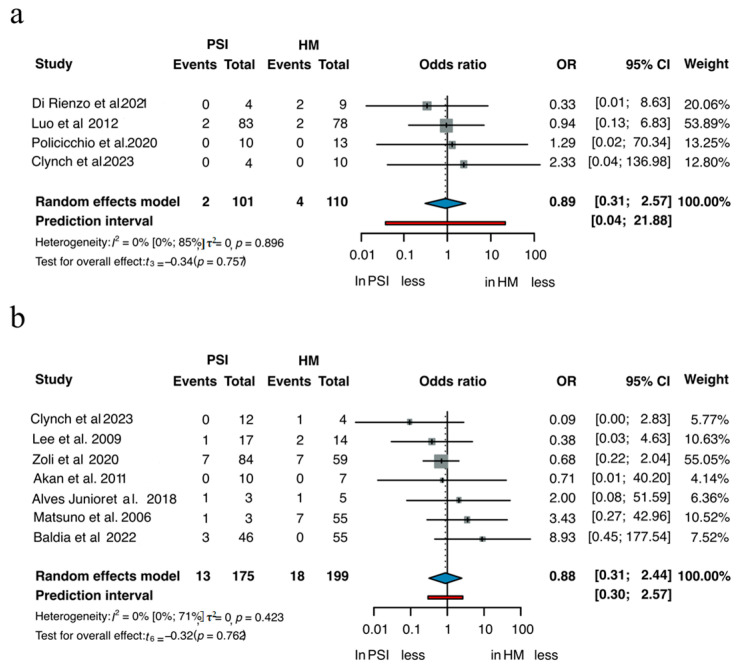
SSI of (**a**) Titanium and (**b**) PMMA [3,22,28,40,45,77,83,88,102,138].

Ten studies enabled direct comparison of SSI between PSIs and HM implants. Subgroup analyses by material are shown in Figure 4 [3,22,28,40,45,77,83,88,102,138]. Among 101 patients treated with PSIs and 110 with HM titanium implants, the OR for SSI was 0.89 in favor of PSIs (95% CI 0.31–2.57, *p* = 0.757). In the PMMA group, which included 175 PSI cases and 199 HM, the OR was 0.88 (95% CI 0.31–2.44, *p* = 0.762). In both comparisons, the differences in infection rates were not statistically significant, although the point estimates slightly favored PSI.

Table S4 summarizes the one-arm analyses of SSI. Infection rates varied across materials, with several PSIs, including CaP-titanium, hydroxyapatite, and titanium, showing postoperative infection probabilities below 6%. PMMA and PEEK PSIs demonstrated infection rates between 8.1% and 9.5%. Among the HM implants, autologous bone and PMMA showed infection rates of 8.7%, while hydroxyapatite reached the highest proportion at 14.9% (Figure 3b).

### 3.5. Total Reoperation

Table S5 summarizes the results of the one-arm analyses for total reoperations. PSIs showed lower reoperation proportions across most materials compared to HM implants (Figure 3c). The lowest PSI reoperation rates were observed for CaP-Ti (4.7%) and Hydroxyapatite (6.7%), while higher rates were found for PEEK (10.6%) and PMMA (10.8%). Among the HM implants, hydroxyapatite, autologous bone and PMMA exhibited reoperation rates of 11%, with titanium reaching the highest value at 12.6%.

### 3.6. Operation Time

Table S6 presents the pooled analysis of operation times across materials. Procedures performed with PSIs were generally shorter than those with HM implants. The mean duration for PSIs ranged from 98 min for hydroxyapatite to 201 min for Porous polyethylene, with titanium and PMMA PSIs averaging around 116 and 125 min, respectively (Figure 3e). In contrast, HM implants required longer operative times, with titanium averaging 151 min, autologous bone 156 min, and PMMA nearly 191 min, representing the longest recorded duration among all materials. These findings highlight a consistent reduction in surgical time when using PSI, particularly for titanium and PMMA, where differences exceeded 35–65 min compared to their HM counterparts.

### 3.7. Cosmetic Score

Table S7 displays the cosmetic outcomes assessed by visual analogue scale (VAS, 0–10, with 10 indicating the highest satisfaction). Across the included studies, PSIs consistently achieved higher cosmetic ratings compared to HM implants (Figure 3d). Mean scores for PSI materials generally ranged between 8.2 and 8.4, reflecting favorable esthetic outcomes across titanium, PMMA, PEEK, and hydroxyapatite reconstructions. By contrast, HM implants more frequently scored between 5.8 and 7.1, indicating moderate satisfaction but a clear reduction compared with PSI. The widest gap was observed for PMMA, where PSI reconstructions approached VAS scores of 8.3, whereas HM PMMA averaged closer to 7.1. Although the mean differences often appear small (1.2 VAS points), this shift typically represents the transition from “acceptable” to “near-perfect” esthetics in CP, where psychosocial reintegration and patient self-image are central, such improvements are clinically meaningful.

### 3.8. Implant Price

Table S8 summarizes the reported implant costs across included studies. Prices varied substantially depending on material and implant type. Among PSI materials, PEEK showed the highest average costs, ranging from approximately USD 14414 to 27902 per implant. Titanium PSIs were reported between USD 5627 and 7858, while PMMA PSIs were substantially lower, with reported prices ranging from USD 398 to 5565. HM titanium implants were less expensive, reported between USD 2143 and 2893.

### 3.9. Certainty of Evidence

The certainty of evidence for the two-arm studies was evaluated using the GRADE system and was overall rated as low. A detailed assessment is presented in Supplementary Material (Supplementary Material Figure S3).

## 4. Discussion

### 4.1. Summary of Key Findings

This study presents the most comprehensive systematic review and meta-analysis to date comparing PSIs and HM implants in CP. Overall, the findings suggest that PSI, regardless of the material used, may offer superior outcomes in terms of surgical efficiency and postoperative complications. Specifically, PSIs were associated with shorter operation times, reduced odds of implant removal and fewer overall secondary operations compared to HM alternatives. These trends were observed consistently across materials such as titanium, PMMA, and hydroxyapatite, highlighting the potential benefits of preoperative customization in cranioplasty procedures. However, these results should be interpreted with caution due to the high heterogeneity among the included studies, including variations in surgical technique, patient populations and follow-up duration. Moreover, none of the single-arm analyses reached statistical significance.

### 4.2. Material-Specific Considerations

Although customized implant technologies involve greater cost and effort, their superiority over conventional HM systems has not been clearly demonstrated, a finding similar to that in several single-center studies [40,99,129,138]. HM PMMA implants gained popularity for decades as a practical and inexpensive option in CP. Their widespread use was driven by immediate intraoperative availability, low material cost, and the relative ease of shaping PMMA directly at the surgical site to match the defect. Several single-center reports emphasized its value as a rapid solution, especially in settings with limited resources or when custom-made prostheses were not accessible [77,138]. Despite these advantages, however, outcomes varied considerably depending on defect size, anatomical location, and the surgeon’s experience, underlining both the appeal and the limitations of this technique [139]. In our analysis, the two-arm comparison favored PSI PMMA, and the meta-regression estimated the explantation probability of HM PMMA at 14.2%. The high rate of explantation seen with HM PMMA can be attributed to its material-specific limitations, particularly residual monomer toxicity arising from intraoperative polymerization. The exothermic reaction and the use of autopolymerizing PMMA, often with suboptimal ratios of monomer to powder, can lead to excess unreacted monomers. These substances have been shown to cause cytotoxic effects, inflammatory responses, and even neurotoxicity when monomers are inadvertently dispersed into the brain during cooling with saline [22]. Moreover, direct contact with the dura and the need for intraoperative shaping and drilling may further increase the risk of foreign body responses, ultimately contributing to implant failure and removal [140]. PEEK was also associated with relatively high rates of both implant removal (8.2%) and SSI (9.5%) compared to other PSI, which likely reflects its frequent use in complex, high-risk reconstructions [23,31]. PEEK is often chosen for more complex or high-risk cases, such as large cranial defects, syndromic conditions, or tumor resections, where PSIs are preferred for their precise fit.

### 4.3. Influence of Surgical and Patient Factors

The timing between craniectomy and CP appears to have a notable influence on postoperative outcomes. Performing CP too early may increase the risk of complications such as infection and inflammation, likely due to residual contamination, incomplete resolution of cerebral edema, or compromised wound healing. Conversely, delayed CP can lead to extensive dural scarring, bone resorption or brain atrophy, which may complicate implant integration [141]. However, according to a recent meta-analysis by Malcolm et al., no significant difference in postoperative complication rates was observed when comparing early (<90 days) versus delayed (>90 days) CP [142]. Anatomical factors may also influence complication rates. Frontal bone defects, which were present in several included studies, are associated with thinner soft tissue coverage, potentially increasing the risk of implant exposure and chronic inflammatory response, particularly when using materials with known toxicity profiles. Moreover, preoperative radiotherapy is a well-documented factor that significantly worsens surgical outcomes, increasing the risk of postoperative complications by up to sevenfold [143]. Radiation alters local vascularity, impairs tissue regeneration, and induces chronic inflammation, all of which can compromise wound healing and promote implant-related complications, including implant failure. These patients typically present with longer operative times and higher complication risks, both contributing to elevated postoperative infection rates [100]. This trend is consistent with our findings of PEEK PSIs having one of the longest average operation times (170.34 min). In contrast, other PSI, such as hydroxyapatite, titanium, and PMMA demonstrated shorter operation times, which is likely due to their precise preoperative planning and optimal fit, reducing the need for intraoperative adjustments. On the other end of the spectrum, HM PMMA showed one of the longest overall operation time (190.54 min), which may be explained by the intraoperative polymerization process and the need for manual sculpting and fitting during surgery, steps that can be time-consuming and technically demanding [22]. The 35–65 min reduction observed with titanium and PMMA PSIs may have broader consequences, including shorter anesthesia duration, reduced intraoperative blood loss, and potentially fewer infection-related complications, which are particularly relevant in critically ill or polytrauma patients [144].

### 4.4. Biological and Biomechanical Factors Underlying Outcomes

Infection rates in our analysis ranged from a postoperative probability of 2.9% to 14.9%, with the highest observed in HM hydroxyapatite implants (14.9%). The elevated infection rate in HM hydroxyapatite may be attributed to challenges in intraoperative handling, increased porosity, and suboptimal fit, all of which can compromise soft tissue closure and increase contamination risk [101]. By contrast, CaP-Ti PSIs demonstrated the lowest infection probability (2.9%), followed by titanium PSIs (5.5%) and PMMA PSIs (8.1%). PEEK PSIs showed relatively high infection rates (9.5%), consistent with its bioinert, non-osseointegrating properties, while autologous bone and PMMA HM also demonstrated elevated risks (8.7% each). When considering overall reoperations, a similar pattern emerged. Beyond infection and revision rates, the biological interaction between implant and host tissue provides further explanation. PEEK, while widely used, is hydrophobic and bioinert, limiting osteoblast adhesion and preventing osseointegration, which may predispose it to implant migration and infection. In contrast, CaP-Ti implants promote neovascularization and bone ingrowth, enhancing stability, wound healing, and even allowing local antibiotic release when pre-soaked in gentamicin [100]. Similarly, hydroxyapatite supports osseointegration, although its brittleness can increase fracture susceptibility [5]. These findings highlight that, in addition to mechanical properties and surgical handling, implant biocompatibility plays a critical role in long-term CP outcomes.

### 4.5. Economic Considerations

Titanium has long been a favored material for CP due to its biocompatibility, strength, and ease of shaping. HM titanium meshes offer a relatively inexpensive solution, with costs reported around USD 1500 for large implants, whereas computer-aided patient-specific titanium implants are typically two to three times more expensive (USD 2700–7800 depending on size and manufacturer). Despite the higher cost, PSIs reduce implant removals and secondary reoperations, potentially offsetting expenses in high-resource healthcare systems, while HM meshes remain attractive in resource-limited settings where affordability is a primary concern [83].

### 4.6. Strengths and Limitations

The main strength of this study lies in its comprehensive scope and methodological detail. By including all major implant materials and explicitly separating patient-specific from HM implants, this analysis provides a more precise and transparent comparison than previous reviews. This approach allows for a clearer understanding of recovery dynamics and the relative effectiveness of different implant types. Furthermore, the breadth of the dataset, which represents the largest synthesis of CP outcomes to date, enhances the robustness of the findings and supports their applicability across a wide clinical spectrum. Despite these strengths, several limitations should be acknowledged. The included studies demonstrated a high degree of heterogeneity, with notable variation in surgical techniques, patient populations, and the way outcomes were reported. In addition, differences in how studies defined and classified events likely introduced further residual heterogeneity. Together, these factors complicate direct comparability across studies. Importantly, nearly all of the available evidence derives from observational studies, with only a single randomized controlled trial included. This reliance on non-randomized data increases the risk of bias and limits the strength of causal inferences. Additionally, the literature provides little clarity on the clinical decision-making process regarding when a PSI or HM implant should be preferred, restricting the ability to draw practice-oriented recommendations. While these limitations do not diminish the relevance of the findings, they emphasize the need for cautious interpretation and for future high-quality prospective studies to establish clearer guidance.

### 4.7. Implications for Practice and Implications for Research

Translating scientific evidence into daily surgical decision-making is essential [145,146]. Where financial resources and technical infrastructure allow, PSIs should be prioritized. Their tailored design improves intraoperative precision, reduces operative time, and lowers the likelihood of complications, making them particularly valuable in complex reconstructions, frontal bone reconstructions with limited soft tissue coverage, or in cases where anatomical accuracy is critical. Conversely, HM implants remain an important option in situations for smaller defects or in emergency situations where custom fabrication is not feasible.

From a research perspective, greater transparency in reporting is required, including the consistent provision of raw outcome data and standardized definitions of complications. Future studies should not only compare patient-specific and HM implants across different materials but also aim to establish clearer criteria for implant selection in clinical practice. Although materials were analyzed separately to avoid inappropriate cross-material pooling, exploring correlations or hierarchical relationships between materials may help identify broader patterns of implant performance. Such analyses represent an important direction for future research.

## 5. Conclusions

Across materials, PSIs were associated with favorable trends in shorter operative time, less explantations, fewer reoperations, and better cosmetic satisfaction compared with HM implants, highlighting the benefits of preoperative customization. However, most data derive from observational cohorts, and many direct comparisons were not statistically significant. Therefore, these findings should be interpreted as associative rather than demonstrating proven superiority. 

## Figures and Tables

**Table 1:** Demographic data of the patients. ^a^ Patient-Specific Implant, ^b^ Hand-molded, NA: Not available, PMMA: Polymethylmethacrylate, AB: Autologous Bone, PEEK: Polyetheretherketone, Ti: Titanium, HA: Hydroxyapatite, PP: Porous polyethylene, CaP-Ti: Calcium Phosphate-Titanium.

First Author and Year	Country	Patient Number and Material	Reported Outcomes for Meta-Analysis		
			Postoperative Complications	Surgery Time	Cosmetic Score
Akan et al., 2011 [22]	Turkey	10 ^a^, 7 ^b^ PMMA	Yes	No	No
Alawi et al., 2024 [23]	Oman	28 AB; 14 PEEK	Yes	No	No
Alves Junior et al., 2018 [3]	Brazil	11 AB; 3 ^a^, 5 ^b^ PMMA	Yes	Yes	No
Amin et al., 2024 [24]	Bangladesh	11 PEEK	Yes	No	No
Anele et al., 2024 [25]	Nigeria	8 ^b^ Ti	Yes	No	No
Anto et al., 2017 [26]	India	72 AB	Yes	No	No
Ashraf et al., 2022 [27]	Pakistan	10 ^a^ PMMA	Yes	No	Yes
Baldia et al., 2022 [28]	India	46 ^a^, 24 ^b^ PMMA	Yes	No	Yes
Basu et al., 2021 [29]	India	10 ^a^ PMMA	Yes	Yes	No
Bianchi et al., 2019 [30]	Italy	6 PEEK	Yes	Yes	No
Binhammer et al., 2020 [2]	Canada	23 ^a^, 25 ^b^ Ti; 26 ^a^ PMMA; 13 AB; 11 PEEK	Yes	Yes	No
Brandicourt et al., 2017 [31]	France	37 PEEK	Yes	No	Yes
Brie et al., 2013 [32]	France	8 ^a^ HA	Yes	Yes	No
Cabraja et al., 2009 [33]	Germany	26 ^a^ Ti	Yes	Yes	No
Caro-Osorio et al., 2013 [34]	Mexico	26 ^a^ PMMA	Yes	Yes	No
Champeaux et al., 2019 [35]	France	19 ^a^ Ti	Yes	No	No
Chen et al., 2015 [36]	Taiwan	7 ^a^ Ti	Yes	Yes	No
Chen et al., 2018 [37]	China	57 ^a^ HA	Yes	No	No
Cheng et al., 2008 [38]	Taiwan	52 AB; 23 ^b^ PMMA	Yes	No	No
Cheng et al., 2018 [39]	Taiwan	10 ^a^ PMMA	Yes	Yes	No
Clynch et al., 2023 [40]	United Kingdom	12 ^a^, 4 ^b^ PMMA; 4 ^a^, 10 ^b^ Ti; 2 ^a^ HA	Yes	No	No
Couldwell et al., 1994 [41]	USA	25 PP	Yes	No	No
Csámer et al., 2023 [42]	Hungary	52 ^a^ PMMA	Yes	No	No
Da Silva Júnior et al., 2021 [43]	Brazil	16 ^a^ PMMA	Yes	No	No
Desai et al., 2019 [44]	India	30 ^a^ PMMA	Yes	Yes	Yes
Di Rienzo et al., 2021 [45]	Italy	4 ^a^, 9 ^b^ Ti; 9 ^a^ PMMA; 5 ^b^ HA; 21 PEEK	Yes	No	No
Duric et al., 2019 [46]	Croatia	29 ^a^ PMMA	Yes	Yes	Yes
Eom et al., 2020 [47]	South Korea	19 ^b^ Ti	Yes	No	No
Eufinger et al., 1998 [48]	Germany	22 ^a^ Ti	Yes	No	No
Fong et al., 2015 [49]	USA	13 AB	Yes	No	No
Fountain et al., 2021 [50]	United Kingdom	35 AB; 17 PEEK; 8 PP	Yes	Yes	No
Francaviglia et al., 2017 [51]	Italy	10 ^a^ Ti	Yes	No	No
Ganau et al., 2020 [52]	France	92 ^a^ HA; 89 ^a^ PMMA	Yes	No	No
Giese et al., 2020 [53]	Germany	67 ^a^ PMMA	Yes	No	Yes
Gilardino et al., 2015 [54]	Canada	7 PEEK	Yes	No	No
Goh et al., 2010 [55]	Taiwan	31 ^a^ PMMA	Yes	No	No
Hamböck et al., 2020 [56]	Austria	119 AB; 37 ^b^ PMMA	Yes	No	No
He et al., 2022 [57]	China	104 PEEK	Yes	No	No
Heissler et al., 1998 [58]	Germany	15 ^a^ Ti	Yes	No	No
Hoffmann et al., 2005 [59]	Germany	15 ^a^ Ti	Yes	Yes	No
Honeybul et al., 2012 [60]	Australia	156 AB	Yes	No	No
Hosameldin et al., 2021 [61]	Egypt	33 PEEK; 33 ^b^ HA	Yes	Yes	Yes
Huang et al., 2015 [62]	USA	20 ^a^ PMMA	Yes	No	No
Iaccarino et al., 2015 [63]	Italy	31 AB; 50 ^a^ HA; 13 ^a^ PMMA; 2 PEEK	Yes	No	No
Inoue et al., 1995 [64]	Japan	8 AB	Yes	No	No
Iratwar et al., 2024 [65]	India	10 ^a^ PMMA	Yes	Yes	No
Jaberi et al., 2013 [66]	USA	70 ^a^ PMMA	Yes	No	No
Jin et al., 2016 [67]	China	39 ^a^ Ti	Yes	Yes	No
Jonkergouw et al., 2016 [68]	Netherlands	38 PEEK	Yes	Yes	No
Kim et al., 2012 [69]	South Korea	16 ^a^ PMMA	Yes	Yes	No
Kim et al., 2018 [70]	South Korea	45 AB; 31 ^a^ Ti; 32 PP	Yes	Yes	Yes
Kim et al., 2023 [71]	South Korea	35 ^a^ Ti	Yes	Yes	No
Kiyokawa et al., 1998 [72]	Japan	12 AB	Yes	No	No
Kohan et al., 2015 [73]	USA	28 AB; 11 ^b^ Ti; 13 PEEK	Yes	Yes	No
Kung et al., 2012a [74]	Taiwan	40 ^a^ Ti	Yes	Yes	No
Kung et al., 2012b [75]	Taiwan	9 ^b^ PMMA	Yes	Yes	No
Kwiecien et al., 2018 [76]	USA	36 AB; 130 ^b^ Ti	Yes	No	No
Lee et al., 2009 [77]	Taiwan	91 AB; 17 ^a^, 23 ^b^ PMMA	Yes	Yes	No
Lee et al., 2012 [78]	South Korea	118 AB	Yes	Yes	No
Lee et al., 2014 [79]	South Korea	18 AB	Yes	No	No
Lemée et al., 2013 [80]	France	5 AB; 7 ^a^ HA	Yes	No	No
Lethaus et al., 2014 [81]	Netherlands	16 AB	Yes	Yes	No
Linder et al., 2019 [82]	Sweden	50 CaP-Ti	Yes	No	No
Lindner et al., 2017 [5]	Germany	24 ^a^ Ti; 26 ^a^ HA	Yes	Yes	No
Luo et al., 2012 [83]	China	83 ^a^, 78 ^b^ Ti	Yes	Yes	No
Maenhoudt et al., 2018 [84]	Belgium	16 ^a^ HA	Yes	No	No
Marbacher et al., 2012 [85]	Switzerland	27 ^a^ PMMA	Yes	No	No
Maricevich et al., 2019 [86]	Brazil	63 ^a^ PMMA	Yes	No	No
Marlier et al., 2017 [87]	France	23 PP	Yes	No	No
Matsuno et al., 2006 [88]	Japan	54 AB; 3 ^a^, 55 ^b^ PMMA; 77 ^a^ Ti	Yes	No	No
Moellmann et al., 2022 [9]	Germany	39 PEEK	Yes	Yes	No
Moles et al., 2018 [89]	France	44 AB; 48 ^a^ HA	Yes	Yes	Yes
Morales-Gómez et al., 2018 [90]	Mexico	22 ^a^ PMMA	Yes	Yes	No
Moreira-Gonzalez et al., 2003 [91]	USA	312 AB; 58 ^b^ HA; 75 ^b^ PMMA	Yes	No	No
Morina et al., 2011 [92]	Kosovo	75 AB	Yes	No	No
Morton et al., 2016 [93]	USA	532 AB; 151 PEEK; 23 PP	Yes	No	No
Moser et al., 2017 [94]	Switzerland	17 ^a^ PMMA	Yes	Yes	No
Mrad et al., 2017 [95]	Canada	10 AB; 9 PEEK	Yes	Yes	No
Nagarjuna et al., 2015 [8]	India	5 ^a^ Ti	Yes	No	No
Ng et al., 2014 [96]	Singapore	7 ^b^ PMMA; 5 ^b^ Ti; 12 PEEK	Yes	Yes	No
Nguyen et al., 2021 [97]	Vietnam	35 ^a^ Ti	Yes	No	No
O Reilly et al., 2015 [98]	USA	19 PEEK	Yes	No	No
Ou et al., 2019 [99]	China	107 AB; 136 ^b^ PMMA	Yes	No	No
Pfnür et al., 2024 [100]	Germany	25 AB; 35 PEEK; 2 ^a^ HA; 21 CaP-Ti	Yes	Yes	No
Piitulainen et al., 2015 [101]	Finland	20 AB; 31 ^b^ HA; 11 ^b^ PMMA	Yes	No	No
Policicchio et al., 2020 [102]	Italy	10 ^a^, 13 ^b^ Ti	Yes	Yes	Yes
Pöppe et al., 2022 [103]	Austria	14 ^a^ PMMA	Yes	Yes	No
Rammos et al., 2015 [104]	USA	11 PEEK	Yes	No	No
Ridwan-Pramana et al., 2019 [105]	Netherlands	16 ^a^ PMMA	Yes	No	No
Rosenthal et al., 2014 [106]	Israel	65 PEEK	Yes	No	No
Rosinski et al., 2020 [107]	USA	21 PEEK; 61 ^b^ Ti	Yes	Yes	No
Rotaru et al., 2012 [108]	Romania	10 ^a^ PMMA	Yes	No	No
Sahoo et al., 2010 [109]	India	11 AB; 6 ^b^ Ti; 5 ^a^ PMMA	Yes	No	No
Sahoo et al., 2019 [110]	India	12 AB	Yes	No	No
Saxena et al., 2023 [7]	India	5 AB; 5 ^a^ Ti; 5 PEEK	Yes	No	No
Schoekler et al., 2014 [111]	Austria	45 AB	Yes	No	No
Schön et al., 2021 [112]	Switzerland	16 ^a^ PMMA	Yes	Yes	No
Sharavanan et al., 2015 [113]	India	29 ^a^ PMMA	Yes	No	No
Shay et al., 2020 [114]	USA	55 ^a^ PMMA	Yes	No	No
Shi et al., 2023 [115]	China	89 ^b^ Ti; 66 CaP-Ti	Yes	No	No
Soto et al., 2022 [116]	USA	27 AB	Yes	No	No
Splavski et al., 2022 [117]	Croatia	5 ^a^ PMMA	Yes	Yes	No
Staffa et al., 2007 [118]	Italy	25 ^a^ HA	Yes	Yes	No
Stefini et al., 2015 [119]	Italy	2489 ^a^ HA	Yes	No	No
Stieglitz et al., 2014 [120]	Switzerland	28 ^a^ PMMA	Yes	No	No
Sun et al., 2019 [121]	China	207 ^a^ Ti	Yes	No	No
Sundseth et al., 2013 [122]	Norway	13 PP	Yes	Yes	No
Tehli et al., 2023 [123]	Turkey	26 ^a^ Ti	Yes	No	No
Tel et al., 2021 [124]	Italy	7 ^a^ PMMA	Yes	No	No
Thien et al., 2015 [125]	Singapore	24 PEEK; 108 ^a^ Ti	Yes	No	No
Unterhofer et al., 2017 [126]	Austria	46 ^a^ PMMA	Yes	No	Yes
Van Gool et al., 1985 [127]	Netherlands	45 ^a^ PMMA	Yes	No	No
Vargo et al., 2020 [128]	USA	11 PEEK; 10 ^b^ Ti	Yes	No	No
Velnar et al., 2022 [129]	Slovenia	12 ^b^ PMMA	Yes	No	No
Vince et al., 2019 [130]	Germany	221 AB; 65 ^b^ PMMA	Yes	No	No
Vlok et al., 2018 [131]	South Africa	30 ^a^ PMMA	Yes	No	No
Wang et al., 2012 [132]	China	23 PP	Yes	No	No
Wesp et al., 2022 [133]	Germany	43 ^a^ PMMA; 39 CaP-Ti	Yes	No	No
Williams et al., 2015 [134]	United Kingdom	149 ^a^ Ti	Yes	No	No
Yao et al., 2022 [135]	China	106 PEEK; 105 ^b^ Ti	Yes	Yes	No
Yoon et al., 2021 [6]	South Korea	40 ^a^ Ti	Yes	Yes	No
Zegers et al., 2017 [1]	Netherlands	8 ^a^ Ti; 21 PEEK	Yes	No	Yes
Zhang et al., 2015 [136]	China	8 ^a^ Ti	Yes	No	No
Zhang et al., 2018 [137]	China	75 PEEK; 110 ^b^ Ti	Yes	Yes	No
Zoli et al., 2020 [138]	Italy	84 ^a^, 59 ^b^ PMMA	Yes	No	No

## Data Availability

The datasets used in this study can be found in the full-text articles included in the systematic review and meta-analysis. If further information is needed, it will be provided upon reasonable request to the corresponding author.

## Supplementary Materials

The following supporting information can be downloaded at: https://www.mdpi.com/article/10.3390/jcm14248655/s1, Text S1: Predefined search key, Figure S1: RoB 2 assessment, Figure S2: ROBINS-I. assessment for (a) implant removal and (b) SSI, Figure S3: GRADE assessment for all subgroups, Table S1: PRISMA Checklist, Table S2: MINORS assessment for single-arm studies, Table S3: Implant removal across all one-arm studies, Table S4: SSI across all one-arm studies, Table S5: Total number of reoperations across all one-arm studies, Table S6: Operation time in minutes, Table S7: Cosmetic Score on VAS (0–10), Table S8: Implant Price in USD.
