# Supplementary material for: Comparative Safety and Efficacy of Patient-Specific Versus Hand-Molded Implants in Cranioplasty: A Systematic Review and Meta-Analysis
Source: J Clin Med. 2025 Dec 6;14(24):8655. doi: 10.3390/jcm14248655 (PMC12733873; doi:10.3390/jcm14248655)
Supplement: Supplementary file 1 [file jcm-14-08655-s001.zip › jcm-4001216-supplementary.pdf]

## Supplementary Material

Text S1. Predefined search key.

Figure S1. RoB 2 assessment.

Figure S2. ROBINS-I assessment for (a) Implant removal and (b) SSI.

Figure S3. GRADE assessment for all subgroups.

Table S1. PRISMA Checklist.

Table S2. MINORS assessment for single-arm studies.

Table S3. Implant removal across all one-arm studies.

Table S4. SSI across all one-arm studies.

Table S5. Total number of reoperations across all one-arm studies.

Table S6. Operation time in minutes.

Table S7. Cosmetic Score on VAS (0-10).

Table S8. Implant Price in USD.

**Text S1. Predefined search key.**

("cranioplasty" OR "cranial" OR "skull" OR "craniofacial" OR "face" OR "calvarial") AND ("reconstruction" OR "implant" OR prosthes* OR "replacement") AND ("alloplastic material" OR "autologous material" OR titanium* OR "PEEK" OR "polyetheretherketone" OR "PMMA" OR "polymethylmethacrylate" OR "bone cement" OR "porous polyethylene" OR "calcium phosphate" OR "vascularized bone" OR "non vascularized bone" OR "CAD" OR "computer aided design" OR "CAM" OR "computer aided manufacturing" OR "custom" OR "patient specific" OR "bone graft" OR "hydroxyapatite" OR "composite" OR "bone matrix" OR "polyethylene")

**Figure S1. RoB 2 assessment.**

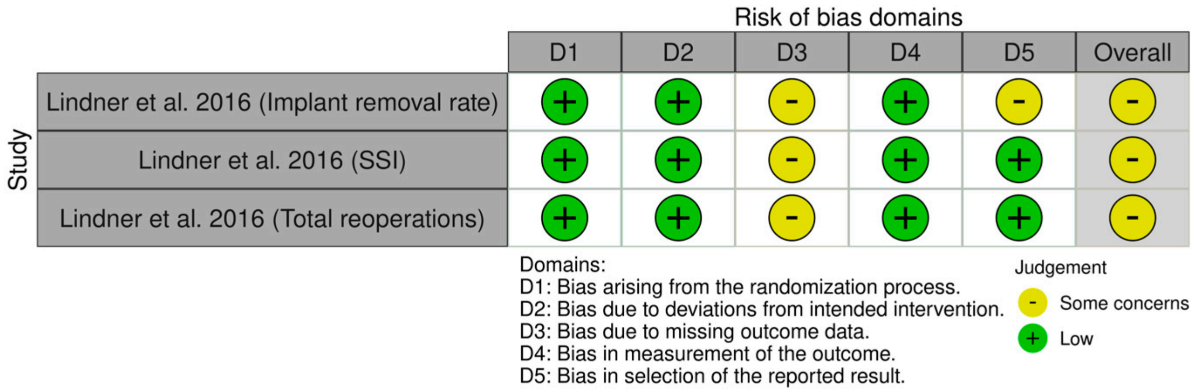

**Figure S2. ROBINS-I assessment for (a) Implant removal and (b) SSI.**

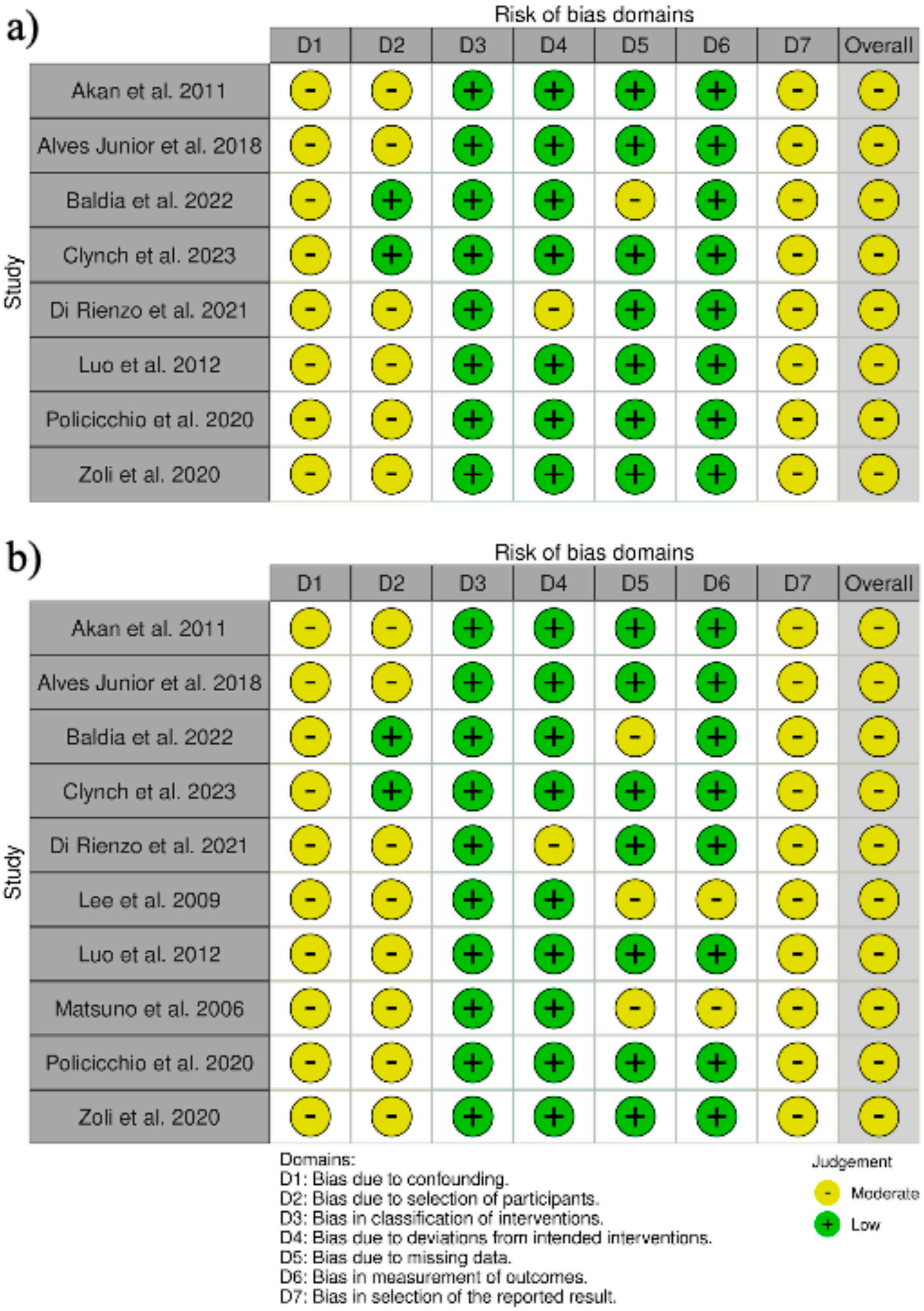

## Figure S3. GRADE assessment for all subgroups.

**Summary of findings:**

### PSI compared to Hand-molded in Cranioplasty

**Patient or population:** Cranioplasty
**Intervention:** PSI
**Comparison:** Hand-molded

| Outcomes | Anticipated absolute effects* (95% CI) Risk with Hand-molded | Risk with PSI | Relative effect (95% CI) | № of participants (studies) | Certainty of the evidence (GRADE) | Comments |
|---|---|---|---|---|---|---|
| Implant removal-Titanium (two-arm) | 64 per 1,000 | **23 per 1,000** (7 to 65) | **OR 0.34** (0.11 to 1.03) | 211 (4 non-randomized studies) | ⨁⨁◯◯ Low | |
| Implant removal-PMMA (two-arm) | 141 per 1,000 | **84 per 1,000** (33 to 202) | **OR 0.56** (0.21 to 1.54) | 254 (5 non-randomized studies) | ⨁⨁◯◯ Low | |
| SSI-Titanium (two-arm) | 36 per 1,000 | **32 per 1,000** (12 to 88) | **OR 0.89** (0.31 to 2.57) | 211 (4 non-randomized studies) | ⨁⨁◯◯ Low | |
| SSI-PMMA (two-arm) | 90 per 1,000 | **80 per 1,000** (30 to 195) | **OR 0.88** (0.31 to 2.44) | 374 (7 non-randomized studies) | ⨁⨁◯◯ Low | |

***The risk in the intervention group** (and its 95% confidence interval) is based on the assumed risk in the comparison group and the **relative effect** of the intervention (and its 95% CI).

**CI:** confidence interval; **OR:** odds ratio

**GRADE Working Group grades of evidence**
**High certainty:** we are very confident that the true effect lies close to that of the estimate of the effect.
**Moderate certainty:** we are moderately confident in the effect estimate: the true effect is likely to be close to the estimate of the effect, but there is a possibility that it is substantially different.
**Low certainty:** our confidence in the effect estimate is limited: the true effect may be substantially different from the estimate of the effect.
**Very low certainty:** we have very little confidence in the effect estimate: the true effect is likely to be substantially different from the estimate of effect.

**Table S1. PRISMA Checklist.**

PRISMA 2020 Checklist

| Section and Topic | Item # | Checklist item | Location where item is reported |
|---|---|---|---|
| **TITLE** | | | |
| Title | 1 | Identify the report as a systematic review. | p.1 |
| **ABSTRACT** | | | |
| Abstract | 2 | See the PRISMA 2020 for Abstracts checklist. | p.1 |
| **INTRODUCTION** | | | |
| Rationale | 3 | Describe the rationale for the review in the context of existing knowledge. | p.2 |
| Objectives | 4 | Provide an explicit statement of the objective(s) or question(s) the review addresses. | p.2 |
| **METHODS** | | | |
| Eligibility criteria | 5 | Specify the inclusion and exclusion criteria for the review and how studies were grouped for the syntheses. | p.3 |
| Information sources | 6 | Specify all databases, registers, websites, organisations, reference lists and other sources searched or consulted to identify studies. Specify the date when each source was last searched or consulted. | p.3 |
| Search strategy | 7 | Present the full search strategies for all databases, registers and websites, including any filters and limits used. | p.3, 5 |
| Selection process | 8 | Specify the methods used to decide whether a study met the inclusion criteria of the review, including how many reviewers screened each record and each report retrieved, whether they worked independently, and if applicable, details of automation tools used in the process. | p.3 |
| Data collection process | 9 | Specify the methods used to collect data from reports, including how many reviewers collected data from each report, whether they worked independently, any processes for obtaining or confirming data from study investigators, and if applicable, details of automation tools used in the process. | p.3 |
| Data items | 10a | List and define all outcomes for which data were sought. Specify whether all results that were compatible with each outcome domain in each study were sought (e.g. for all measures, time points, analyses), and if not, the methods used to decide which results to collect. | p.3 |
| | 10b | List and define all other variables for which data were sought (e.g. participant and intervention characteristics, funding sources). Describe any assumptions made about any missing or unclear information. | p.3 |
| Study risk of bias assessment | 11 | Specify the methods used to assess risk of bias in the included studies, including details of the tool(s) used, how many reviewers assessed each study and whether they worked independently, and if applicable, details of automation tools used in the process. | p.3, 4 |
| Effect measures | 12 | Specify for each outcome the effect measure(s) (e.g. risk ratio, mean difference) used in the synthesis or presentation of results. | p.4 |
| Synthesis methods | 13a | Describe the processes used to decide which studies were eligible for each synthesis (e.g. tabulating the study intervention characteristics and comparing against the planned groups for each synthesis (item #5)). | p.4, 5 |
| | 13b | Describe any methods required to prepare the data for presentation or synthesis, such as handling of missing summary statistics, or data conversions. | p.5 |
| | 13c | Describe any methods used to tabulate or visually display results of individual studies and syntheses. | p.5 |
| | 13d | Describe any methods used to synthesize results and provide a rationale for the choice(s). If meta-analysis was performed, describe the model(s), method(s) to identify the presence and extent of statistical heterogeneity, and software package(s) used. | p.5 |
| | 13e | Describe any methods used to explore possible causes of heterogeneity among study results (e.g. subgroup analysis, meta-regression). | p.5 |
| | 13f | Describe any sensitivity analyses conducted to assess robustness of the synthesized results. | p.5 |
| Reporting bias assessment | 14 | Describe any methods used to assess risk of bias due to missing results in a synthesis (arising from reporting biases). | p.5 |
| Certainty assessment | 15 | Describe any methods used to assess certainty (or confidence) in the body of evidence for an outcome. | p.5 |

PRISMA 2020 Checklist

| Section and Topic | Item # | Checklist item | Location where item is reported |
|---|---|---|---|
| **RESULTS** | | | |
| Study selection | 16a | Describe the results of the search and selection process, from the number of records identified in the search to the number of studies included in the review, ideally using a flow diagram. | p.4 |
| | 16b | Cite studies that might appear to meet the inclusion criteria, but which were excluded, and explain why they were excluded. | p.4-5 |
| Study characteristics | 17 | Cite each included study and present its characteristics. | p.4-11 |
| Risk of bias in studies | 18 | Present assessments of risk of bias for each included study. | Supplement. Material |
| Results of individual studies | 19 | For all outcomes, present, for each study: (a) summary statistics for each group (where appropriate) and (b) an effect estimate and its precision (e.g. confidence/credible interval), ideally using structured tables or plots. | p.12-18 |
| Results of syntheses | 20a | For each synthesis, briefly summarise the characteristics and risk of bias among contributing studies. | p.12-18 |
| | 20b | Present results of all statistical syntheses conducted. If meta-analysis was done, present for each the summary estimate and its precision (e.g. confidence/credible interval) and measures of statistical heterogeneity. If comparing groups, describe the direction of the effect. | p.12-18 |
| | 20c | Present results of all investigations of possible causes of heterogeneity among study results. | p.12-18 |
| | 20d | Present results of all sensitivity analyses conducted to assess the robustness of the synthesized results. | p.12-18 |
| Reporting biases | 21 | Present assessments of risk of bias due to missing results (arising from reporting biases) for each synthesis assessed. | p.12 |
| Certainty of evidence | 22 | Present assessments of certainty (or confidence) in the body of evidence for each outcome assessed. | p.18 |
| **DISCUSSION** | | | |
| Discussion | 23a | Provide a general interpretation of the results in the context of other evidence. | p.18-21 |
| | 23b | Discuss any limitations of the evidence included in the review. | p.20 |
| | 23c | Discuss any limitations of the review processes used. | p.20 |
| | 23d | Discuss implications of the results for practice, policy, and future research. | p.21 |
| **OTHER INFORMATION** | | | |
| Registration and protocol | 24a | Provide registration information for the review, including register name and registration number, or state that the review was not registered. | p.2 |
| | 24b | Indicate where the review protocol can be accessed, or state that a protocol was not prepared. | p.2 |
| | 24c | Describe and explain any amendments to information provided at registration or in the protocol. | p.2 |
| Support | 25 | Describe sources of financial or non-financial support for the review, and the role of the funders or sponsors in the review. | p.27 |
| Competing interests | 26 | Declare any competing interests of review authors. | p.28 |
| Availability of data, code and other materials | 27 | Report which of the following are publicly available and where they can be found: template data collection forms; data extracted from included studies; data used for all analyses; analytic code; any other materials used in the review. | p.28 |

**Table S2. MINORS assessment for single-arm studies.** D1: A clearly stated aim; D2: Inclusion of consecutive patients; D3: Prospective collection of data; D4: Endpoints appropriate to the aim of the study; D5: Unbiased assessment of the study endpoint; D6: Follow-up period appropriate to the aim of the study; D7: Loss to follow-up less than 5%; D8: Prospective calculation of the study size. According to the MINORS criteria, scores of 0–8 indicate low, 9–12 moderate, and 13–16 high methodological quality for non-comparative studies.

| Study | D1 | D2 | D3 | D4 | D5 | D6 | D7 | D8 | Overall |
|---|---|---|---|---|---|---|---|---|---|
| SSI | | | | | | | | | |
| Al Alawi et al. 2024 | 2 | 1 | 0 | 2 | 1 | 2 | 2 | 0 | 10 |
| Amin et al. 2024 | 2 | 1 | 0 | 2 | 1 | 2 | 2 | 0 | 10 |
| Anele et al. 2024 | 2 | 1 | 0 | 2 | 1 | 2 | 2 | 0 | 10 |
| Anto et al. 2017 | 2 | 1 | 0 | 2 | 1 | 2 | 2 | 0 | 10 |
| Ashraf et al. 2022 | 2 | 1 | 0 | 2 | 1 | 2 | 2 | 0 | 10 |
| Basu et al. 2021 | 2 | 1 | 0 | 2 | 1 | 2 | 2 | 0 | 10 |
| Bianchi et al. 2019 | 2 | 1 | 0 | 2 | 1 | 2 | 2 | 0 | 10 |
| Binhammer et al. 2019 | 2 | 1 | 0 | 2 | 1 | 2 | 2 | 0 | 10 |
| Brandicourt et al. 2017 | 2 | 1 | 0 | 2 | 1 | 2 | 1 | 0 | 9 |
| Brie et al. 2013 | 2 | 1 | 0 | 2 | 1 | 2 | 1 | 0 | 9 |
| Caro-Osorio et al. 2013 | 2 | 1 | 0 | 2 | 1 | 2 | 2 | 0 | 10 |
| Chen et al. 2015 | 2 | 1 | 0 | 2 | 1 | 2 | 2 | 0 | 10 |
| Chen et al. 2018 | 2 | 1 | 0 | 2 | 1 | 2 | 1 | 0 | 9 |
| Cheng et al. 2008 | 2 | 1 | 0 | 2 | 1 | 2 | 2 | 0 | 10 |
| Cheng et al. 2018 | 2 | 1 | 0 | 2 | 1 | 2 | 2 | 0 | 10 |
| Couldwell et al. 1994 | 2 | 1 | 0 | 2 | 1 | 2 | 2 | 0 | 10 |
| Csámer et al. 2023 | 2 | 1 | 0 | 2 | 1 | 2 | 2 | 0 | 10 |
| Da Silva Júnior et al. 2021 | 2 | 1 | 0 | 2 | 1 | 2 | 2 | 0 | 10 |
| Desai et al. 2019 | 2 | 1 | 0 | 2 | 1 | 2 | 2 | 0 | 10 |
| Eom et al. 2020 | 2 | 1 | 0 | 2 | 1 | 2 | 2 | 0 | 10 |
| Fountain et al. 2021 | 2 | 1 | 2 | 2 | 1 | 2 | 2 | 0 | 12 |
| Francaviglia et al. 2017 | 2 | 1 | 0 | 2 | 1 | 2 | 2 | 0 | 10 |
| Ganau et al. 2020 | 2 | 1 | 0 | 2 | 1 | 2 | 2 | 0 | 10 |
| Giese et al. 2020 | 2 | 1 | 0 | 2 | 1 | 2 | 2 | 0 | 10 |
| Gilardino et al. 2015 | 2 | 1 | 0 | 2 | 1 | 2 | 2 | 0 | 10 |

| | | | | | | | | | | |
|---|---|---|---|---|---|---|---|---|---|---|
| Goh et al. 2010 | 2 | 1 | 0 | 2 | 1 | 2 | 1 | 0 | 9 | |
| Hamböck et al. 2020 | 2 | 1 | 0 | 2 | 1 | 2 | 2 | 0 | 10 | |
| He et al. 2022 | 2 | 1 | 0 | 2 | 1 | 2 | 1 | 0 | 9 | |
| Heissler et al. 1998 | 2 | 1 | 0 | 2 | 1 | 2 | 2 | 0 | 10 | |
| Hoffmann et al. 2005 | 2 | 1 | 0 | 2 | 1 | 2 | 2 | 0 | 10 | |
| Honeybul et al. 2012 | 2 | 1 | 0 | 2 | 1 | 2 | 2 | 0 | 10 | |
| Hosameldin et al. 2021 | 2 | 1 | 0 | 2 | 1 | 2 | 1 | 0 | 9 | |
| Huang et al. 2015 | 2 | 1 | 0 | 2 | 1 | 2 | 2 | 0 | 10 | 1 |
| Iaccarino et al. 2015 | 2 | 1 | 2 | 2 | 1 | 2 | 1 | 0 | 11 | |
| Inoue et al. 1995 | 2 | 1 | 0 | 2 | 1 | 2 | 2 | 0 | 10 | |
| Iratwar et al. 2024 | 2 | 1 | 0 | 2 | 1 | 2 | 2 | 0 | 10 | |
| Jaberi et al. 2013 | 2 | 1 | 0 | 2 | 1 | 2 | 1 | 0 | 9 | |
| Jin et al. 2016 | 2 | 1 | 0 | 2 | 1 | 2 | 2 | 0 | 10 | |
| Jonkergouw et al. 2016 | 2 | 1 | 0 | 2 | 1 | 2 | 2 | 0 | 10 | |
| Kim et al. 2012 | 2 | 1 | 0 | 2 | 1 | 2 | 2 | 0 | 10 | |
| Kim et al. 2018 | 2 | 1 | 0 | 2 | 1 | 2 | 2 | 0 | 10 | |
| Kim et al. 2023 | 2 | 1 | 0 | 2 | 1 | 2 | 2 | 0 | 10 | |
| Kiyokawa et al. 1998 | 2 | 1 | 0 | 2 | 1 | 2 | 2 | 0 | 10 | |
| Kohan et al. 2015 | 2 | 1 | 0 | 2 | 1 | 2 | 1 | 0 | 9 | |
| KungA et al. 2012 | 2 | 1 | 0 | 2 | 1 | 2 | 2 | 0 | 10 | |
| KungB et al. 2012 | 2 | 1 | 0 | 2 | 1 | 2 | 2 | 0 | 10 | |
| Kwiecien et al. 2018 | 2 | 1 | 0 | 2 | 1 | 2 | 2 | 0 | 10 | |
| Lee et al. 2012 | 2 | 1 | 0 | 2 | 1 | 2 | 2 | 0 | 10 | |
| Lee et al. 2014 | 2 | 1 | 0 | 2 | 1 | 2 | 2 | 0 | 10 | |
| Linder et al. 2019 | 2 | 1 | 0 | 2 | 1 | 2 | 2 | 0 | 10 | 1 |
| Maenhoudt et al. 2018 | 2 | 1 | 0 | 2 | 1 | 2 | 2 | 0 | 10 | |
| Marbacher et al. 2012 | 2 | 1 | 0 | 2 | 1 | 2 | 1 | 0 | 9 | |
| Maricevich et al. 2019 | 2 | 1 | 2 | 2 | 1 | 2 | 1 | 0 | 11 | |
| Marlier et al. 2017 | 2 | 1 | 0 | 2 | 1 | 2 | 2 | 0 | 10 | |
| Moellmann et al. 2022 | 2 | 1 | 0 | 2 | 1 | 2 | 2 | 0 | 10 | |
| Moles et al. 2018 | 2 | 1 | 0 | 2 | 1 | 2 | 2 | 0 | 10 | |
| Morales-Gómez et al. 2018 | 2 | 1 | 0 | 2 | 1 | 2 | 2 | 0 | 10 | |
| Moreira-Gonzalez et al. 2003 | 2 | 1 | 0 | 2 | 1 | 2 | 2 | 0 | 10 | |
| Morina et al. 2011 | 2 | 1 | 2 | 2 | 1 | 2 | 2 | 0 | 12 | |

| | | | | | | | | | | |
|---|---|---|---|---|---|---|---|---|---|---|
| Morton et al. 2016 | 2 | 1 | 0 | 2 | 1 | 2 | 2 | 0 | 10 | |
| Moser et al. 2017 | 2 | 1 | 0 | 2 | 1 | 2 | 0 | 0 | 8 | |
| Nagarjuna et al. 2015 | 2 | 1 | 0 | 2 | 1 | 2 | 2 | 0 | 10 | |
| Ng et al. 2014 | 2 | 1 | 0 | 2 | 1 | 2 | 2 | 0 | 10 | |
| Nguyen et al. 2021 | 2 | 1 | 0 | 2 | 1 | 2 | 2 | 0 | 10 | |
| O Reilly et al. 2015 | 2 | 1 | 0 | 2 | 1 | 2 | 2 | 0 | 10 | |
| Ou et al. 2019 | 2 | 1 | 0 | 2 | 1 | 2 | 1 | 0 | 9 | |
| Pfnür et al. 2024 | 2 | 1 | 0 | 2 | 1 | 2 | 2 | 0 | 10 | 1 |
| Piitulainen et al. 2015 | 2 | 1 | 0 | 2 | 1 | 2 | 0 | 0 | 8 | |
| Pöppe et al. 2022 | 2 | 1 | 0 | 2 | 1 | 2 | 2 | 0 | 10 | |
| Rammos et al. 2015 | 2 | 1 | 0 | 2 | 1 | 2 | 2 | 0 | 10 | |
| Rosenthal et al. 2014 | 2 | 1 | 0 | 2 | 1 | 2 | 0 | 0 | 8 | |
| Rosinski et al. 2020 | 2 | 1 | 0 | 2 | 1 | 2 | 1 | 0 | 9 | |
| Rotaru et al. 2012 | 2 | 1 | 0 | 2 | 1 | 2 | 2 | 0 | 10 | |
| Sahoo et al. 2010 | 2 | 1 | 0 | 2 | 1 | 2 | 2 | 0 | 10 | |
| Sahoo et al. 2019 | 2 | 1 | 0 | 2 | 1 | 2 | 2 | 0 | 10 | |
| Saxena et al. 2023 | 2 | 1 | 2 | 2 | 1 | 2 | 2 | 0 | 12 | |
| Schoekler et al. 2014 | 2 | 1 | 0 | 2 | 1 | 2 | 1 | 0 | 9 | |
| Schön et al. 2021 | 2 | 1 | 0 | 2 | 1 | 2 | 2 | 0 | 10 | |
| Shay et al. 2020 | 2 | 1 | 0 | 2 | 1 | 2 | 2 | 0 | 10 | |
| Shi et al. 2023 | 2 | 1 | 0 | 2 | 1 | 2 | 1 | 0 | 9 | |
| Soto et al. 2022 | 2 | 1 | 0 | 2 | 1 | 2 | 1 | 0 | 9 | |
| Splavski et al. 2022 | 2 | 1 | 0 | 2 | 1 | 2 | 2 | 0 | 10 | |
| Staffa et al. 2007 | 2 | 1 | 0 | 2 | 1 | 2 | 2 | 0 | 10 | |
| Stefini et al. 2015 | 2 | 1 | 0 | 2 | 1 | 2 | 1 | 0 | 9 | 1 |
| Stieglitz et al. 2014 | 2 | 1 | 0 | 2 | 1 | 2 | 2 | 0 | 10 | |
| Sun et al. 2019 | 2 | 1 | 2 | 2 | 1 | 2 | 2 | 0 | 12 | |
| Sundseth et al. 2013 | 2 | 1 | 0 | 2 | 1 | 2 | 2 | 0 | 10 | |
| Tel et al. 2021 | 2 | 1 | 0 | 2 | 1 | 2 | 2 | 0 | 10 | |
| Thien et al. 2015 | 2 | 1 | 0 | 2 | 1 | 2 | 1 | 0 | 9 | |
| Unterhofer et al. 2017 | 2 | 1 | 0 | 2 | 1 | 2 | 2 | 0 | 10 | |
| Van Gool et al. 1985 | 2 | 1 | 0 | 2 | 1 | 2 | 1 | 0 | 9 | 1 |
| Vargo et al. 2020 | 2 | 1 | 0 | 2 | 1 | 2 | 2 | 0 | 10 | |
| Velnar et al. 2022 | 2 | 1 | 0 | 2 | 1 | 2 | 2 | 0 | 10 | |
| Vince et al. 2019 | 2 | 1 | 0 | 2 | 1 | 2 | 2 | 0 | 10 | |
| Vlok et al. 2018 | 2 | 1 | 2 | 2 | 1 | 2 | 2 | 0 | 12 | |
| Wang et al. 2012 | 2 | 1 | 0 | 2 | 1 | 2 | 2 | 0 | 10 | |
| Wesp et al. 2022 | 2 | 1 | 0 | 2 | 1 | 2 | 1 | 0 | 9 | |
| Williams et al. 2015 | 2 | 1 | 0 | 2 | 1 | 2 | 2 | 0 | 10 | 1 |

| | | | | | | | | | | |
|---|---|---|---|---|---|---|---|---|---|---|
| Yao et al. 2022 | 2 | 1 | 0 | 2 | 1 | 2 | 1 | 0 | 9 | |
| Yoon et al. 2021 | 2 | 1 | 0 | 2 | 1 | 2 | 2 | 0 | 10 | |
| Zegers et al. 2017 | 2 | 1 | 0 | 2 | 1 | 2 | 1 | 0 | 9 | |
| Zhang et al. 2015 | 2 | 1 | 0 | 2 | 1 | 2 | 2 | 0 | 10 | |
| Zhang et al. 2018 | 2 | 1 | 0 | 2 | 1 | 2 | 2 | 0 | 10 | |
| Implant removal | | | | | | | | | | |
| Amin et al. 2024 | 2 | 1 | 0 | 2 | 1 | 2 | 2 | 0 | 10 | |
| Anele et al. 2024 | 2 | 1 | 0 | 2 | 1 | 2 | 2 | 0 | 10 | |
| Anto et al. 2017 | 2 | 1 | 0 | 2 | 1 | 2 | 2 | 0 | 10 | |
| Ashraf et al. 2022 | 2 | 1 | 0 | 2 | 1 | 2 | 2 | 0 | 10 | |
| Basu et al. 2021 | 2 | 1 | 0 | 2 | 1 | 2 | 2 | 0 | 10 | |
| Bianchi et al. 2019 | 2 | 1 | 0 | 2 | 1 | 2 | 2 | 0 | 10 | |
| Brandicourt et al. 2017 | 2 | 1 | 0 | 2 | 1 | 2 | 1 | 0 | 9 | |
| Brie et al. 2013 | 2 | 1 | 0 | 2 | 1 | 2 | 1 | 0 | 9 | |
| Caro-Osorio et al. 2013 | 2 | 1 | 0 | 2 | 1 | 2 | 2 | 0 | 10 | |
| Chen et al. 2015 | 2 | 1 | 0 | 2 | 1 | 2 | 2 | 0 | 10 | |
| Cheng et al. 2008 | 2 | 1 | 0 | 2 | 1 | 2 | 2 | 0 | 10 | |
| Cheng et al. 2018 | 2 | 1 | 0 | 2 | 1 | 2 | 2 | 0 | 10 | |
| Csámer et al. 2023 | 2 | 1 | 0 | 2 | 1 | 2 | 2 | 0 | 10 | |
| da Silva Júnior et al. 2021 | 2 | 1 | 0 | 2 | 1 | 2 | 2 | 0 | 10 | |
| Desai et al. 2019 | 2 | 1 | 0 | 2 | 1 | 2 | 2 | 0 | 10 | |
| Eom et al. 2020 | 2 | 1 | 0 | 2 | 1 | 2 | 2 | 0 | 10 | |
| Francaviglia et al. 2017 | 2 | 1 | 0 | 2 | 1 | 2 | 2 | 0 | 10 | |
| Ganau et al. 2020 | 2 | 1 | 0 | 2 | 1 | 2 | 2 | 0 | 10 | |
| Giese et al. 2020 | 2 | 1 | 0 | 2 | 1 | 2 | 2 | 0 | 10 | |
| Gilardino et al. 2015 | 2 | 1 | 0 | 2 | 1 | 2 | 2 | 0 | 10 | |
| Goh et al. 2010 | 2 | 1 | 0 | 2 | 1 | 2 | 1 | 0 | 9 | |
| Hamböck et al. 2020 | 2 | 1 | 0 | 2 | 1 | 2 | 2 | 0 | 10 | |
| Heissler et al. 1998 | 2 | 1 | 0 | 2 | 1 | 2 | 2 | 0 | 10 | |
| Hoffmann et al. 2005 | 2 | 1 | 0 | 2 | 1 | 2 | 2 | 0 | 10 | |
| Honeybul et al. 2012 | 2 | 1 | 0 | 2 | 1 | 2 | 2 | 0 | 10 | |
| Hosameldin et al. 2021 | 2 | 1 | 0 | 2 | 1 | 2 | 1 | 0 | 9 | |
| Huang et al. 2015 | 2 | 1 | 0 | 2 | 1 | 2 | 2 | 0 | 10 | 1 |
| Iaccarino et al. 2015 | 2 | 1 | 2 | 2 | 1 | 2 | 1 | 0 | 11 | |
| Inoue et al. 1995 | 2 | 1 | 0 | 2 | 1 | 2 | 2 | 0 | 10 | |
| Iratwar et al. 2024 | 2 | 1 | 0 | 2 | 1 | 2 | 2 | 0 | 10 | |
| Jaberi et al. 2013 | 2 | 1 | 0 | 2 | 1 | 2 | 1 | 0 | 9 | |

| | | | | | | | | | | |
|---|---|---|---|---|---|---|---|---|---|---|
| Jonkergouw et al. 2016 | 2 | 1 | 0 | 2 | 1 | 2 | 2 | 0 | 10 | |
| Kim et al. 2012 | 2 | 1 | 0 | 2 | 1 | 2 | 2 | 0 | 10 | |
| Kim et al. 2018 | 2 | 1 | 0 | 2 | 1 | 2 | 2 | 0 | 10 | |
| Kiyokawa et al. 1998 | 2 | 1 | 0 | 2 | 1 | 2 | 2 | 0 | 10 | |
| Kohan et al. 2015 | 2 | 1 | 0 | 2 | 1 | 2 | 1 | 0 | 9 | |
| Kwiecien et al. 2018 | 2 | 1 | 0 | 2 | 1 | 2 | 2 | 0 | 10 | |
| Lee et al. 2012 | 2 | 1 | 0 | 2 | 1 | 2 | 2 | 0 | 10 | |
| Lee et al. 2014 | 2 | 1 | 0 | 2 | 1 | 2 | 2 | 0 | 10 | |
| Linder et al. 2019 | 2 | 1 | 0 | 2 | 1 | 2 | 2 | 0 | 10 | 1 |
| Maenhoudt et al. 2018 | 2 | 1 | 0 | 2 | 1 | 2 | 2 | 0 | 10 | |
| Marbacher et al. 2012 | 2 | 1 | 0 | 2 | 1 | 2 | 1 | 0 | 9 | |
| Maricevich et al. 2019 | 2 | 1 | 2 | 2 | 1 | 2 | 1 | 0 | 11 | |
| Marlier et al. 2017 | 2 | 1 | 0 | 2 | 1 | 2 | 2 | 0 | 10 | |
| Moellmann et al. 2022 | 2 | 1 | 0 | 2 | 1 | 2 | 2 | 0 | 10 | |
| Moles et al. 2018 | 2 | 1 | 0 | 2 | 1 | 2 | 2 | 0 | 10 | |
| Morales-Gómez et al. 2018 | 2 | 1 | 0 | 2 | 1 | 2 | 2 | 0 | 10 | |
| Morina et al. 2011 | 2 | 1 | 2 | 2 | 1 | 2 | 2 | 0 | 12 | |
| Moser et al. 2017 | 2 | 1 | 0 | 2 | 1 | 2 | 0 | 0 | 8 | |
| Nagarjuna et al. 2015 | 2 | 1 | 0 | 2 | 1 | 2 | 2 | 0 | 10 | |
| Ng et al. 2014 | 2 | 1 | 0 | 2 | 1 | 2 | 2 | 0 | 10 | |
| Nguyen et al. 2021 | 2 | 1 | 0 | 2 | 1 | 2 | 2 | 0 | 10 | |
| O Reilly et al. 2015 | 2 | 1 | 0 | 2 | 1 | 2 | 2 | 0 | 10 | |
| Pfnür et al. 2024 | 2 | 1 | 0 | 2 | 1 | 2 | 2 | 0 | 10 | 1 |
| Piitulainen et al. 2015 | 2 | 1 | 0 | 2 | 1 | 2 | 0 | 0 | 8 | |
| Pöppe et al. 2022 | 2 | 1 | 0 | 2 | 1 | 2 | 2 | 0 | 10 | |
| Rammos et al. 2015 | 2 | 1 | 0 | 2 | 1 | 2 | 2 | 0 | 10 | |
| Rosenthal et al. 2014 | 2 | 1 | 0 | 2 | 1 | 2 | 0 | 0 | 8 | |
| Rosinski et al. 2020 | 2 | 1 | 0 | 2 | 1 | 2 | 1 | 0 | 9 | |
| Rotaru et al. 2012 | 2 | 1 | 0 | 2 | 1 | 2 | 2 | 0 | 10 | |
| Sahoo et al. 2010 | 2 | 1 | 0 | 2 | 1 | 2 | 2 | 0 | 10 | |
| Sahoo et al. 2019 | 2 | 1 | 0 | 2 | 1 | 2 | 2 | 0 | 10 | |
| Saxena et al. 2023 | 2 | 1 | 2 | 2 | 1 | 2 | 2 | 0 | 12 | |
| Schön et al. 2021 | 2 | 1 | 0 | 2 | 1 | 2 | 2 | 0 | 10 | |
| Sharavanan et al. 2015 | 2 | 1 | 0 | 2 | 1 | 2 | 2 | 0 | 10 | |

| | | | | | | | | | | |
|---|---|---|---|---|---|---|---|---|---|---|
| Shay et al. 2020 | 2 | 1 | 0 | 2 | 1 | 2 | 2 | 0 | 10 | |
| Splavski et al. 2022 | 2 | 1 | 0 | 2 | 1 | 2 | 2 | 0 | 10 | |
| Staffa et al. 2007 | 2 | 1 | 0 | 2 | 1 | 2 | 2 | 0 | 10 | |
| Stieglitz et al. 2014 | 2 | 1 | 0 | 2 | 1 | 2 | 2 | 0 | 10 | |
| Sun et al. 2019 | 2 | 1 | 2 | 2 | 1 | 2 | 2 | 0 | 12 | |
| Sundseth et al. 2013 | 2 | 1 | 0 | 2 | 1 | 2 | 2 | 0 | 10 | |
| Tehli et al. 2023 | 2 | 1 | 0 | 2 | 1 | 2 | 2 | 0 | 10 | |
| Tel et al. 2021 | 2 | 1 | 0 | 2 | 1 | 2 | 2 | 0 | 10 | |
| Thien et al. 2015 | 2 | 1 | 0 | 2 | 1 | 2 | 1 | 0 | 9 | |
| Unterhofer et al. 2017 | 2 | 1 | 0 | 2 | 1 | 2 | 2 | 0 | 10 | |
| Van Gool et al. 1985 | 2 | 1 | 0 | 2 | 1 | 2 | 1 | 0 | 9 | 1 |
| Vargo et al. 2020 | 2 | 1 | 0 | 2 | 1 | 2 | 2 | 0 | 10 | |
| Velnar et al. 2022 | 2 | 1 | 0 | 2 | 1 | 2 | 2 | 0 | 10 | |
| Wang et al. 2012 | 2 | 1 | 0 | 2 | 1 | 2 | 2 | 0 | 10 | |
| Wesp et al. 2022 | 2 | 1 | 0 | 2 | 1 | 2 | 1 | 0 | 9 | |
| Williams et al. 2015 | 2 | 1 | 0 | 2 | 1 | 2 | 2 | 0 | 10 | 1 |
| Yao et al. 2022 | 2 | 1 | 0 | 2 | 1 | 2 | 1 | 0 | 9 | |
| Yoon et al. 2021 | 2 | 1 | 0 | 2 | 1 | 2 | 2 | 0 | 10 | |
| Zegers et al. 2017 | 2 | 1 | 0 | 2 | 1 | 2 | 1 | 0 | 9 | |
| Zhang et al. 2015 | 2 | 1 | 0 | 2 | 1 | 2 | 2 | 0 | 10 | |
| Zhang et al. 2018 | 2 | 1 | 0 | 2 | 1 | 2 | 2 | 0 | 10 | |
| Operation time | | | | | | | | | | |
| Amin et al. 2024 | 2 | 1 | 0 | 2 | 1 | 2 | 2 | 0 | 10 | |
| Binhammer et al. 2020 | 2 | 1 | 0 | 2 | 1 | 2 | 2 | 0 | 10 | |
| Brandicourt et al. 2017 | 2 | 1 | 0 | 2 | 1 | 2 | 1 | 0 | 9 | |
| Brie et al. 2013 | 2 | 1 | 0 | 2 | 1 | 2 | 1 | 0 | 9 | |
| Cabraja et al. 2009 | 2 | 1 | 0 | 2 | 1 | 2 | 1 | 0 | 9 | |
| Chen et al. 2015 | 2 | 1 | 0 | 2 | 1 | 2 | 2 | 0 | 10 | |
| Chen et al. 2018 | 2 | 1 | 0 | 2 | 1 | 2 | 1 | 0 | 9 | |
| Cheng et al. 2008 | 2 | 1 | 0 | 2 | 1 | 2 | 2 | 0 | 10 | |
| Da Silva Júnior et al. 2021 | 2 | 1 | 0 | 2 | 1 | 2 | 2 | 0 | 10 | |
| Eufinger et al. 1998 | 2 | 1 | 0 | 2 | 1 | 2 | 2 | 0 | 10 | |
| Giese et al. 2020 | 2 | 1 | 0 | 2 | 1 | 2 | 2 | 0 | 10 | |
| He et al. 2022 | 2 | 1 | 0 | 2 | 1 | 2 | 1 | 0 | 9 | |
| Hosameldin et al. 2021 | 2 | 1 | 0 | 2 | 1 | 2 | 1 | 0 | 9 | |
| Iaccarino et al. 2015 | 2 | 1 | 2 | 2 | 1 | 2 | 1 | 0 | 11 | |
| Kim et al. 2012 | 2 | 1 | 0 | 2 | 1 | 2 | 2 | 0 | 10 | |

| | | | | | | | | | | |
|---|---|---|---|---|---|---|---|---|---|---|
| Kim et al. 2018 | 2 | 1 | 0 | 2 | 1 | 2 | 2 | 0 | 10 | |
| Kim et al. 2023 | 2 | 1 | 0 | 2 | 1 | 2 | 2 | 0 | 10 | |
| Kiyokawa et al. 1998 | 2 | 1 | 0 | 2 | 1 | 2 | 2 | 0 | 10 | |
| Kohan et al. 2015 | 2 | 1 | 0 | 2 | 1 | 2 | 1 | 0 | 9 | |
| KungA et al. 2012 | 2 | 1 | 0 | 2 | 1 | 2 | 2 | 0 | 10 | |
| KungB et al. 2012 | 2 | 1 | 0 | 2 | 1 | 2 | 2 | 0 | 10 | |
| Kwiecien et al. 2018 | 2 | 1 | 0 | 2 | 1 | 2 | 2 | 0 | 10 | |
| Lee et al. 2014 | 2 | 1 | 0 | 2 | 1 | 2 | 2 | 0 | 10 | |
| Maenhoudt et al. 2018 | 2 | 1 | 0 | 2 | 1 | 2 | 2 | 0 | 10 | |
| Marbacher et al. 2012 | 2 | 1 | 0 | 2 | 1 | 2 | 1 | 0 | 9 | |
| Maricevich et al. 2019 | 2 | 1 | 2 | 2 | 1 | 2 | 1 | 0 | 11 | |
| Morales-Gómez et al. 2018 | 2 | 1 | 0 | 2 | 1 | 2 | 2 | 0 | 10 | |
| Moreira-Gonzalez et al. 2003 | 2 | 1 | 0 | 2 | 1 | 2 | 2 | 0 | 10 | |
| Morton et al. 2016 | 2 | 1 | 0 | 2 | 1 | 2 | 2 | 0 | 10 | |
| Mrad et al. 2017 | 2 | 1 | 0 | 2 | 1 | 2 | 2 | 0 | 10 | |
| Nagarjuna et al. 2015 | 2 | 1 | 0 | 2 | 1 | 2 | 2 | 0 | 10 | |
| Ng et al. 2014 | 2 | 1 | 0 | 2 | 1 | 2 | 2 | 0 | 10 | |
| Ou et al. 2019 | 2 | 1 | 0 | 2 | 1 | 2 | 1 | 0 | 9 | |
| Pfnür et al. 2024 | 2 | 1 | 0 | 2 | 1 | 2 | 2 | 0 | 10 | 1 |
| Pöppe et al. 2022 | 2 | 1 | 0 | 2 | 1 | 2 | 2 | 0 | 10 | |
| Rosinski et al. 2020 | 2 | 1 | 0 | 2 | 1 | 2 | 1 | 0 | 9 | |
| Schoekler et al. 2014 | 2 | 1 | 0 | 2 | 1 | 2 | 1 | 0 | 9 | |
| Shay et al. 2020 | 2 | 1 | 0 | 2 | 1 | 2 | 2 | 0 | 10 | |
| Shi et al. 2023 | 2 | 1 | 0 | 2 | 1 | 2 | 1 | 0 | 9 | |
| Staffa et al. 2007 | 2 | 1 | 0 | 2 | 1 | 2 | 2 | 0 | 10 | |
| Sun et al. 2019 | 2 | 1 | 2 | 2 | 1 | 2 | 2 | 0 | 12 | |
| Van Gool et al. 1985 | 2 | 1 | 0 | 2 | 1 | 2 | 1 | 0 | 9 | 1 |
| Vargo et al. 2020 | 2 | 1 | 0 | 2 | 1 | 2 | 2 | 0 | 10 | |
| Wang et al. 2012 | 2 | 1 | 0 | 2 | 1 | 2 | 2 | 0 | 10 | |
| Wesp et al. 2022 | 2 | 1 | 0 | 2 | 1 | 2 | 1 | 0 | 9 | |
| Yao et al. 2022 | 2 | 1 | 0 | 2 | 1 | 2 | 1 | 0 | 9 | |
| Yoon et al. 2021 | 2 | 1 | 0 | 2 | 1 | 2 | 2 | 0 | 10 | |
| Zhang et al. 2018 | 2 | 1 | 0 | 2 | 1 | 2 | 2 | 0 | 10 | |
| Total reoperation | | | | | | | | | | |
| Al Alawi et al. 2024 | 2 | 1 | 0 | 2 | 1 | 2 | 2 | 0 | 10 | |
| Amin et al. 2024 | 2 | 1 | 0 | 2 | 1 | 2 | 2 | 0 | 10 | |

| | | | | | | | | |
|---|---|---|---|---|---|---|---|---|---|
| Anele et al. 2024 | 2 | 1 | 0 | 2 | 1 | 2 | 2 | 0 | 10 |
| Anto et al. 2017 | 2 | 1 | 0 | 2 | 1 | 2 | 2 | 0 | 10 |
| Ashraf et al. 2022 | 2 | 1 | 0 | 2 | 1 | 2 | 2 | 0 | 10 |
| Basu et al. 2021 | 2 | 1 | 0 | 2 | 1 | 2 | 2 | 0 | 10 |
| Bianchi et al. 2019 | 2 | 1 | 0 | 2 | 1 | 2 | 2 | 0 | 10 |
| Brie et al. 2013 | 2 | 1 | 0 | 2 | 1 | 2 | 1 | 0 | 9 |
| Caro-Osorio et al. 2013 | 2 | 1 | 0 | 2 | 1 | 2 | 2 | 0 | 10 |
| Chen et al. 2015 | 2 | 1 | 0 | 2 | 1 | 2 | 2 | 0 | 10 |
| Chen et al. 2018 | 2 | 1 | 0 | 2 | 1 | 2 | 1 | 0 | 9 |
| Cheng et al. 2008 | 2 | 1 | 0 | 2 | 1 | 2 | 2 | 0 | 10 |
| Cheng et al. 2018 | 2 | 1 | 0 | 2 | 1 | 2 | 2 | 0 | 10 |
| Csámer et al. 2023 | 2 | 1 | 0 | 2 | 1 | 2 | 2 | 0 | 10 |
| Da Silva Júnior et al. 2021 | 2 | 1 | 0 | 2 | 1 | 2 | 2 | 0 | 10 |
| Desai et al. 2019 | 2 | 1 | 0 | 2 | 1 | 2 | 2 | 0 | 10 |
| Eom et al. 2020 | 2 | 1 | 0 | 2 | 1 | 2 | 2 | 0 | 10 |
| Francaviglia et al. 2017 | 2 | 1 | 0 | 2 | 1 | 2 | 2 | 0 | 10 |
| Ganau et al. 2020 | 2 | 1 | 0 | 2 | 1 | 2 | 2 | 0 | 10 |
| Giese et al. 2020 | 2 | 1 | 0 | 2 | 1 | 2 | 2 | 0 | 10 |
| Gilardino et al. 2015 | 2 | 1 | 0 | 2 | 1 | 2 | 2 | 0 | 10 |
| Goh et al. 2010 | 2 | 1 | 0 | 2 | 1 | 2 | 1 | 0 | 9 |
| Hamböck et al. 2020 | 2 | 1 | 0 | 2 | 1 | 2 | 2 | 0 | 10 |
| Heissler et al. 1998 | 2 | 1 | 0 | 2 | 1 | 2 | 2 | 0 | 10 |
| Hoffmann et al. 2005 | 2 | 1 | 0 | 2 | 1 | 2 | 2 | 0 | 10 |
| Honeybul et al. 2012 | 2 | 1 | 0 | 2 | 1 | 2 | 2 | 0 | 10 |
| Hosameldin et al. 2021 | 2 | 1 | 0 | 2 | 1 | 2 | 1 | 0 | 9 |
| Huang et al. 2015 | 2 | 1 | 0 | 2 | 1 | 2 | 2 | 0 | 10 |
| Iaccarino et al. 2015 | 2 | 1 | 2 | 2 | 1 | 2 | 1 | 0 | 11 |
| Inoue et al. 1995 | 2 | 1 | 0 | 2 | 1 | 2 | 2 | 0 | 10 |
| Iratwar et al. 2024 | 2 | 1 | 0 | 2 | 1 | 2 | 2 | 0 | 10 |
| Jaberi et al. 2013 | 2 | 1 | 0 | 2 | 1 | 2 | 1 | 0 | 9 |
| Jonkergouw et al. 2016 | 2 | 1 | 0 | 2 | 1 | 2 | 2 | 0 | 10 |
| Kim et al. 2012 | 2 | 1 | 0 | 2 | 1 | 2 | 2 | 0 | 10 |
| Kim et al. 2018 | 2 | 1 | 0 | 2 | 1 | 2 | 2 | 0 | 10 |
| Kim et al. 2023 | 2 | 1 | 0 | 2 | 1 | 2 | 2 | 0 | 10 |
| Kiyokawa et al. 1998 | 2 | 1 | 0 | 2 | 1 | 2 | 2 | 0 | 10 |
| Kohan et al. 2015 | 2 | 1 | 0 | 2 | 1 | 2 | 1 | 0 | 9 |
| Kwiecien et al. 2018 | 2 | 1 | 0 | 2 | 1 | 2 | 2 | 0 | 10 |

1

| | | | | | | | | | | |
|---|---|---|---|---|---|---|---|---|---|---|
| Lee et al. 2012 | 2 | 1 | 0 | 2 | 1 | 2 | 2 | 0 | 10 | |
| Lee et al. 2014 | 2 | 1 | 0 | 2 | 1 | 2 | 2 | 0 | 10 | |
| Linder et al. 2019 | 2 | 1 | 0 | 2 | 1 | 2 | 2 | 0 | 10 | 1 |
| Maenhoudt et al. 2018 | 2 | 1 | 0 | 2 | 1 | 2 | 2 | 0 | 10 | |
| Marbacher et al. 2012 | 2 | 1 | 0 | 2 | 1 | 2 | 1 | 0 | 9 | |
| Maricevich et al. 2019 | 2 | 1 | 2 | 2 | 1 | 2 | 1 | 0 | 11 | |
| Marlier et al. 2017 | 2 | 1 | 0 | 2 | 1 | 2 | 2 | 0 | 10 | |
| Moellmann et al. 2022 | 2 | 1 | 0 | 2 | 1 | 2 | 2 | 0 | 10 | |
| Moles et al. 2018 | 2 | 1 | 0 | 2 | 1 | 2 | 2 | 0 | 10 | |
| Morales-Gómez et al. 2018 | 2 | 1 | 0 | 2 | 1 | 2 | 2 | 0 | 10 | |
| Moreira-Gonzalez et al. 2003 | 2 | 1 | 0 | 2 | 1 | 2 | 2 | 0 | 10 | |
| Morina et al. 2011 | 2 | 1 | 2 | 2 | 1 | 2 | 2 | 0 | 12 | |
| Morton et al. 2016 | 2 | 1 | 0 | 2 | 1 | 2 | 2 | 0 | 10 | |
| Moser et al. 2017 | 2 | 1 | 0 | 2 | 1 | 2 | 0 | 0 | 8 | |
| Nagarjuna et al. 2015 | 2 | 1 | 0 | 2 | 1 | 2 | 2 | 0 | 10 | |
| Ng et al. 2014 | 2 | 1 | 0 | 2 | 1 | 2 | 2 | 0 | 10 | |
| Nguyen et al. 2021 | 2 | 1 | 0 | 2 | 1 | 2 | 2 | 0 | 10 | |
| O Reilly et al. 2015 | 2 | 1 | 0 | 2 | 1 | 2 | 2 | 0 | 10 | |
| Pfnür et al. 2024 | 2 | 1 | 0 | 2 | 1 | 2 | 2 | 0 | 10 | 1 |
| Piitulainen et al. 2015 | 2 | 1 | 0 | 2 | 1 | 2 | 0 | 0 | 8 | |
| Pöppe et al. 2022 | 2 | 1 | 0 | 2 | 1 | 2 | 2 | 0 | 10 | |
| Rammos et al. 2015 | 2 | 1 | 0 | 2 | 1 | 2 | 2 | 0 | 10 | |
| Rosenthal et al. 2014 | 2 | 1 | 0 | 2 | 1 | 2 | 0 | 0 | 8 | |
| Rosinski et al. 2020 | 2 | 1 | 0 | 2 | 1 | 2 | 1 | 0 | 9 | |
| Rotaru et al. 2012 | 2 | 1 | 0 | 2 | 1 | 2 | 2 | 0 | 10 | |
| Sahoo et al. 2010 | 2 | 1 | 0 | 2 | 1 | 2 | 2 | 0 | 10 | |
| Sahoo et al. 2019 | 2 | 1 | 0 | 2 | 1 | 2 | 2 | 0 | 10 | |
| Saxena et al. 2023 | 2 | 1 | 2 | 2 | 1 | 2 | 2 | 0 | 12 | |
| Schön et al. 2021 | 2 | 1 | 0 | 2 | 1 | 2 | 2 | 0 | 10 | |
| Shay et al. 2020 | 2 | 1 | 0 | 2 | 1 | 2 | 2 | 0 | 10 | |
| Splavski et al. 2022 | 2 | 1 | 0 | 2 | 1 | 2 | 2 | 0 | 10 | |
| Staffa et al. 2007 | 2 | 1 | 0 | 2 | 1 | 2 | 2 | 0 | 10 | |
| Stieglitz et al. 2014 | 2 | 1 | 0 | 2 | 1 | 2 | 2 | 0 | 10 | |
| Sun et al. 2019 | 2 | 1 | 2 | 2 | 1 | 2 | 2 | 0 | 12 | |
| Sundseth et al. 2013 | 2 | 1 | 0 | 2 | 1 | 2 | 2 | 0 | 10 | |

| | | | | | | | | | | |
|---|---|---|---|---|---|---|---|---|---|---|
| Tel et al. 2021 | 2 | 1 | 0 | 2 | 1 | 2 | 2 | 0 | 10 | |
| Thien et al. 2015 | 2 | 1 | 0 | 2 | 1 | 2 | 1 | 0 | 9 | |
| Unterhofer et al. 2017 | 2 | 1 | 0 | 2 | 1 | 2 | 2 | 0 | 10 | |
| Van Gool et al. 1985 | 2 | 1 | 0 | 2 | 1 | 2 | 1 | 0 | 9 | 1 |
| Vargo et al. 2020 | 2 | 1 | 0 | 2 | 1 | 2 | 2 | 0 | 10 | |
| Velnar et al. 2022 | 2 | 1 | 0 | 2 | 1 | 2 | 2 | 0 | 10 | |
| Vince et al. 2019 | 2 | 1 | 0 | 2 | 1 | 2 | 2 | 0 | 10 | |
| Vlok et al. 2018 | 2 | 1 | 2 | 2 | 1 | 2 | 2 | 0 | 12 | |
| Wang et al. 2012 | 2 | 1 | 0 | 2 | 1 | 2 | 2 | 0 | 10 | |
| Wesp et al. 2022 | 2 | 1 | 0 | 2 | 1 | 2 | 1 | 0 | 9 | |
| Williams et al. 2015 | 2 | 1 | 0 | 2 | 1 | 2 | 2 | 0 | 10 | 1 |
| Yao et al. 2022 | 2 | 1 | 0 | 2 | 1 | 2 | 1 | 0 | 9 | |
| Yoon et al. 2021 | 2 | 1 | 0 | 2 | 1 | 2 | 2 | 0 | 10 | |
| Zegers et al. 2017 | 2 | 1 | 0 | 2 | 1 | 2 | 1 | 0 | 9 | |
| Zhang et al. 2015 | 2 | 1 | 0 | 2 | 1 | 2 | 2 | 0 | 10 | |
| Zhang et al. 2018 | 2 | 1 | 0 | 2 | 1 | 2 | 2 | 0 | 10 | |

**Table S3. Implant removal across all one-arm studies.** PSI: Patient-Specific Implant, HM: Hand-molded, PMMA: Polymethylmethacrylate, PEEK: Polyetheretherketone, CaP-Ti: Calcium Phosphate-Titanium, n= number.

| **Material** | **Probability** | **95 % Confidence Interval of probability** | **Removal (n)** | **Patient (n)** | **Study (n)** |
|---|---|---|---|---|---|
| Porous polyethylene (HM) | 0.05 | 0.017-0.14 | 4 | 91 | 4 |
| CaP-Ti (PSI) | 0.05 | 0.019-0.124 | 8 | 110 | 3 |
| Hydroxyapatite (PSI) | 0.054 | 0.025-0.111 | 7 | 287 | 11 |
| Titanium (PSI) | 0.061 | 0.038-0.095 | 51 | 842 | 22 |
| PMMA (PSI) | 0.079 | 0.057-0.109 | 67 | 976 | 38 |
| PEEK (PSI) | 0.082 | 0.055-0.121 | 45 | 587 | 21 |
| Titanium (HM) | 0.099 | 0.062-0.154 | 33 | 575 | 14 |
| Autologous Bone (HM) | 0.104 | 0.073-0.144 | 94 | 916 | 22 |
| Hydroxyapatite (HM) | 0.108 | 0.046-0.236 | 10 | 69 | 3 |

| | | | | | |
|---|---|---|---|---|---|
| PMMA (HM) | 0.142 | 0.083-0.232 | 26 | 189 | 10 |

**Table S4. SSI across all one-arm studies.** PSI: Patient-Specific Implant, HM: Hand-molded, PMMA: Polymethylmethacrylate, PEEK: Polyetheretherketone, CaP-Ti: Calcium Phosphate-Titanium, n: number.

| Material | Probability | 95 % Confidence Interval of probability | SSI (n) | Patient (n) | Study (n) |
|---|---|---|---|---|---|
| CaP-Ti (PSI) | 0.029 | 0.008-0.095 | 3 | 110 | 3 |
| Hydroxyapatite (PSI) | 0.05 | 0.031-0.081 | 68 | 2884 | 13 |
| Titanium (PSI) | 0.055 | 0.037-0.081 | 42 | 981 | 24 |
| Porous Polyethylene (HM) | 0.056 | 0.025-0.124 | 6 | 147 | 7 |
| Titanium (HM) | 0.078 | 0.05-0.119 | 54 | 664 | 15 |
| PMMA (PSI) | 0.081 | 0.06-0.108 | 81 | 1113 | 40 |
| Autologous Bone (HM) | 0.087 | 0.067-0.112 | 182 | 2363 | 31 |
| PMMA (HM) | 0.087 | 0.056-0.131 | 37 | 552 | 16 |
| PEEK (PSI) | 0.095 | 0.068-0.131 | 72 | 898 | 27 |
| Hydroxyapatite (HM) | 0.149 | 0.065-0.305 | 69 | 11 | 3 |

**Table S5. Total number of reoperations across all one-arm studies.** PSI: Patient-Specific Implant, HM: Hand-molded, PMMA: Polymethylmethacrylate, PEEK: Polyetheretherketone, CaP-Ti: Calcium Phosphate-Titanium, n: number.

| Material | Probability | 95 % Confidence Interval of probability | Reoperation (n) | Patient (n) | Study (n) |
|---|---|---|---|---|---|
| CaP-Ti (PSI) | 0.047 | 0.017-0.121 | 14 | 110 | 3 |

| | | | | | |
|---|---|---|---|---|---|
| Porous polyethylene (HM) | 0.064 | 0.022-0.174 | 5 | 114 | 5 |
| Hydroxyapatite (PSI) | 0.067 | 0.035-0.124 | 27 | 402 | 13 |
| Titanium (PSI) | 0.082 | 0.051-0.128 | 75 | 896 | 24 |
| PEEK (PSI) | 0.106 | 0.07-0.158 | 91 | 763 | 24 |
| PMMA (PSI) | 0.108 | 0.078-0.149 | 128 | 1167 | 41 |
| Hydroxyapatite (HM) | 0.11 | 0.041-0.262 | 10 | 69 | 3 |
| Autologous Bone (HM) | 0.113 | 0.079-0.159 | 202 | 2113 | 28 |
| PMMA (HM) | 0.122 | 0.072-0.199 | 46 | 329 | 12 |
| Titanium (HM) | 0.126 | 0.077-0.2 | 53 | 575 | 14 |

**Table S6. Operation time in minutes.** PSI: Patient-Specific Implant, HM: Hand-molded, PMMA: Polymethylmethacrylate, PEEK: Polyetheretherketone, CaP-Ti: Calcium Phosphate-Titanium, n: number, min: minutes.

| **Material** | **Mean (min)** | **95 % Confidence Interval** | **Patient (n)** | **Study (n)** |
|---|---|---|---|---|
| Hydroxyapatite (PSI) | 97.64 | 59.86-135.41 | 109 | 5 |
| Titanium (PSI) | 115.6 | 96.09-135.1 | 373 | 12 |
| PMMA (PSI) | 124.76 | 108.87-140.64 | 251 | 14 |
| Titanium (HM) | 150.13 | 126.66-173.59 | 408 | 8 |
| Autologous Bone (HM) | 155.02 | 134.61-175.42 | 956 | 11 |
| PEEK (PSI) | 170.34 | 140.45-200.23 | 415 | 12 |
| PMMA (HM) | 190.54 | 155.03-226.05 | 44 | 4 |
| CaP-Ti (PSI) | 200.8 | 88.89-312.71 | 21 | 1 |
| Porous polyethylene (HM) | 201.2 | 87.05-315.35 | 53 | 3 |

**Table S7. Cosmetic Score on VAS (0-10).** PSI: Patient-Specific Implant, HM: Hand-molded, PMMA: Polymethylmethacrylate, PEEK: Polyetheretherketone, CaP-Ti: Calcium Phosphate-Titanium, VAS: Visual Analogue Scale.

| Material | Mean (0-10) | 95 % Confidence Interval | Patient (n) | Study (n) |
|---|---|---|---|---|
| Hydroxyapatite (HM) | 5.76 | 3.04-8.47 | 33 | 1 |
| Porous polyethylene (HM) | 6.25 | 3.25-9.25 | 32 | 1 |
| Autologous Bone (HM) | 6.59 | 4.56-8.61 | 89 | 2 |
| Titanium (HM) | 6.8 | 0.09-13.51 | 13 | 1 |
| PMMA (HM) | 7.14 | 4.54-9.74 | 24 | 1 |
| Titanium (PSI) | 8.22 | 6.39-10.06 | 41 | 2 |
| PEEK (PSI) | 8.25 | 4.85-11.65 | 56 | 2 |
| PMMA (PSI) | 8.3 | 7.37-9.24 | 256 | 7 |
| Hydroxyapatite (PSI) | 8.4 | 5.58-11.26 | 48 | 1 |

**Table S8. Implant Price in USD.** PSI: Patient-Specific Implant, HM: Hand-molded, PMMA: Polymethylmethacrylate, PEEK: Polyetheretherketone, USD: US-Dollar.

| Study | Mean Implant Price in USD |
|---|---|
| PEEK (PSI) | |
| Brandicourt et al. 2017 | 14921.47 |
| Mrad et al. 2017 | 27902.64 |
| O Reilly et al. 2015 | 14414.09 |
| Zhang et al. 2018 | 23810.20 |
| PMMA (PSI) | |
| Caro-Osorio et al. 2013 | 1267.86 |
| Ganau et al. 2020 | 5565.59 |
| Morales-Gómez et al. 2019 | 398.48 |
| Pöppe et al. 2021 | 447.67 |
| Titanium (PSI) | |
| Cabraja et al. 2009 | 7339.70 |
| Kim et al. 2018 | 5627.82 |
| Policicchio et al. 2020 | 7858.36 |
| Titanium (HM) | |

| | |
|---|---|
| Policicchio et al. 2020 | 2143.19 |
| Zhang et al. 2018 | 2893.81 |
